# Seven‐year‐olds' references to internal states when playing with toy figures and a video game

**DOI:** 10.1002/icd.2223

**Published:** 2021-03-09

**Authors:** Salim Hashmi, Amy L. Paine, Dale F. Hay

**Affiliations:** ^1^ Department of Psychology Institute of Psychiatry, Psychology and Neuroscience, King's College London London UK; ^2^ School of Psychology Cardiff University Cardiff UK

**Keywords:** internal state language, middle childhood, play, social understanding, video games

## Abstract

References to internal states (e.g., thoughts, feelings, and desires) indicate children's appreciation of people's inner worlds. Many children spend time playing video games; however, the nature of children's speech when doing so has received little attention. We investigated the use of internal state language (ISL) as 251 seven‐year‐olds played with toy figures and a video game designed for the study. Although children used ISL more when playing with toy figures, children used ISL in both contexts, highlighting video game play as a context where children demonstrate their appreciation of inner worlds. Children's speech in the two contexts differed in how ISL was used: references to children's own internal states were more common when playing the video game, and the characters' internal states more common when playing with the toy figures. These findings are discussed with reference to the format of the play activities affording different opportunities to discuss internal states.

**Highlights:**

1. In traditional play children refer to internal states, however, it is unclear whether this occurs when they play video games.
2. Children referred to internal states when playing with toy figures and a video game, but did so more with the toys.
3. Children's video game play can be used as a new context for the study of children's social understanding.

## 1 INTRODUCTION

Playing video games is a popular way in which children spend their free time (Ofcom, 2019), and is considered as a new way of playing that can complement traditional types of play (Lillard, 2014; Singer & Singer, 2005). However, the nature of children's engagement with video games in addition to traditional play with toys is largely unexplored. This is particularly important given the negative representation of video games in the news coverage (Copenhaver, Mitrofan, & Ferguson, 2017) that may have impeded investigations about positive aspects of video gaming (Bormann & Greitemeyer, 2015). During traditional play, children often use internal state language (ISL) to refer to the thoughts, feelings, and desires of themselves and others, which provides an insight into their appreciation of people's internal worlds of themselves and of others (Carpendale & Lewis, 2015). However, a question remains as to whether children also use ISL when playing in virtual environments (de Rosnay & Hughes, 2006). We examined children's spontaneous references to internal states while playing a video game and when playing with toy figures.

## 2 CHILDREN'S ISL DURING PLAY

Children's propensity to refer to internal states affords insight into their appreciation of the minds of themselves and others; as such, ISL is considered an important marker of children's developing social understanding (Carpendale & Lewis, 2015). ISL is a key feature of children's conversations within their close relationships, for example, with parents (Tompkins, Benigno, Kiger Lee, & Wright, 2018), and with siblings and friends (Leach, Howe, & DeHart, 2017, 2019), which is associated with social understanding in later childhood (Hughes & Dunn, 1998; Ruffman, Slade, & Crowe, 2002). Children's play is a particularly rich context for ISL (Howe, Abuhatoum, & Chang‐Kredl, 2014; Leach, Howe, & Dehart, 2015; Leslie, 1987).

In social pretend play, children refer to internal states in their invitations to commence play, in their construction of shared meanings, negotiation of roles and enactments in the play scenario, and when managing conflicting ideas (Howe, 1991; Leach et al., 2017). Children also refer to internal states in private speech when playing alone (Paine et al., 2019a; Davis, Meins, & Fernyhough, 2013; Krafft & Berk, 1998), when narrating stories (Longobardi, Spataro, Renna, & Rossi‐Arnaud, 2014), and during play with imaginary companions (Davis, Meins, & Fernyhough, 2014). In both social and solitary play, children refer to the inner states of characters in the pretend world (i.e., within the *play frame*; Kane & Furth, 1993), including fictional characters (Scarlett & Wolf, 1979; Wolf & Grollman, 1982) and toys (Paine et al., 2019a; Leach et al., 2017). Referring to other people's internal states, both real and fictional, is an important aspect of children's developing social understanding, as children may come to understand the minds of others through simulating their inner states (Bartsch & Wellman, 1995; Goldman, 2006; Harris, 2000).

## 3 CHILDREN'S SPEECH WHEN PLAYING VIDEO GAMES

It is not clear, however, whether ISL is also used when children play video games, which in middle childhood are played on a regular basis. In the United Kingom, 62% of 5‐to 7‐year‐olds play games on media devices for just over 6 hr a week, and 79% of 8‐ to 11‐year‐olds do so for 9.5 hr a week (Ofcom, 2019). Evidence is mixed as to whether and how children's video game play influences their behaviour, and it is likely any effects are associated with the types of games played and characteristics of the children themselves (see Halbrook, O'Donnell, & Msetfi, 2019). Evidence is mixed regarding the influence of violent video game content on behaviour (Anderson & Bushman, 2018), although a recent meta‐analysis did not demonstrate links between violent video games and long‐term youth aggression (Drummond, Sauer, & Ferguson, 2020). Other evidence suggests children's aggressive choices in video game play can be predicted by well‐known risk factors for aggressive conduct problems and angry aggressiveness in infancy (Hay et al., 2017), and engaging with video games prosocially is positively associated with empathy and prosocial behaviour (Harrington & O'Connell, 2016). However, there remain few studies that focus on the positive features children's engagement with video game play (Bormann & Greitemeyer, 2015; Singer & Singer, 2013).

Children talk to themselves, that is, use private speech, while playing video games, as they do during free play (Søndergaard, 2013), and it is likely they would refer to internal states of characters in their speech while playing video games. Children themselves regard playing video games as an imaginative activity, similar to playing with toys (Downey, Hayes, & O'Neill, 2007). In common with traditional play, video game play takes place within fictional worlds (Lillard, 2013), and can evoke emotional reactions (Cairns, Cox, & Nordin, 2014), which reflect *engagement*, *absorption*, or *immersion* within the game's fictional world (Brown & Cairns, 2004; Harris, 2000; Liao & Gendler, 2011). Qualitative research highlights children's imagination as critical for becoming immersed in a virtual world (Søndergaard, 2013). Indeed, 6‐ to 8‐year‐olds make references to the cognitive states of computers (e.g., what computers “*think*” and “*know*”; Turkle, 1997). Children also refer to electronic toys, such as Tamagotchi or Furby, as possessing internal states (e.g., “he's *sad*” and “he *wants* it”; Francis & Mishra, 2009) and treat such toys as if they are living entities, more so than traditional toys (Plowman & Luckin, 2004; Subrahmanyam, Kraut, Greenfield, & Gross, 2001).

Although children may produce ISL in both contexts of play, video games and play with toys have different inherent properties and demands that may affect the nature of children's ISL. Differences in play materials and task structure are known to affect children's play (Trawick‐Smith, Russell, & Swaminathan, 2010), including elements of their speech (Paine et al., 2019b; Krafft & Berk, 1998). For example, props such as toys and dress‐up clothes prompt social interaction and imagination more so than more structured toys and activities, such as maths games and puzzles (Trawick‐Smith et al., 2010).

We hypothesized that these inherent differences in the two types of play may influence the *amount* of ISL, the *category* of internal state that is referred to (e.g., cognitions, emotions, desires, etc.), and/or the *referent* (e.g., the child, the character, etc.) of the internal state being mentioned (Longobardi et al., 2014). Free play with toys is “open‐ended,” with children unconstrained in what they do. In contrast, video games are often “close‐ended,” analogous to games‐with‐rules, where players are often given a series of challenges to complete the game.

Furthermore, the two types of play offer the child opportunities to take on different roles. In video games, children often experience the game from one perspective, that of a “virtual self” or *avatar* (Klimmt, Hefner, & Vorderer, 2009). In contrast, in traditional play with toy figures, children can engage in play as an actor, narrator, or manager of the play (Scarlett & Wolf, 1979). When acting, children are in the role of a character, whereas when narrating or managing (i.e., setting up the toys), children adopt a perspective that is “out” of the play (Giffin, 1984; Scarlett & Wolf, 1979). These different ways of playing are associated with pretend play and different uses of ISL. Playing with toys in expected ways is positively associated with referring to *cognitions* and *beliefs*, and playing with toys in creative ways is associated with using these terms as well as pretence in play (Howe et al., 2014; Howe & Bruno, 2010), whereas simply setting up objects and toys is associated with less pretence in play and fewer references to preferences and beliefs (Howe et al., 2014; Howe & Bruno, 2010).

Despite expecting differences in children's speech across the two types of play, we also hypothesized there would be some consistency in the use of ISL when playing with toy figures and when playing video games. Children's use of ISL has been shown to be consistent over time (Carr, Slade, Yuill, Sullivan, & Ruffman, 2018) and across different contexts (Longobardi et al., 2014). However, such consistency could be explained by other child‐related factors associated with children's ability to understand the minds of others. For example, it is well established that children's age and verbal ability are related to markers of children's social understanding, including their ISL in conversation (de Rosnay & Hughes, 2006; Slade & Ruffman, 2005). Children's higher‐order processes that control thought and behaviour (i.e., executive functioning, such as working memory) share a similar developmental timetable to children's developing social understanding, and are related to social understanding skills in early to middle childhood (Paine et al., 2019a; Bock, Gallaway, & Hund, 2015; Carlson & Moses, 2001; Devine & Hughes, 2014).

The family environment is also important. Children in a family at a socioeconomic disadvantage may have less opportunity for the types of discourse (e.g., reflective discourse) that promote the development of social understanding (Cole & Mitchell, 2000). In addition, children's early conversational environment, in terms of their caregivers' references to mental states, is associated with children's social understanding (Ensor, Devine, Marks, & Hughes, 2014; Meins et al., 2002). It is also possible that children's verbal engagement with each context of play, as well as their existing preferences and experience with different types of toys and technology, may influence their propensity to reflect on inner states. Therefore, we tested whether these characteristics of the child and family were associated with the use of ISL in virtual environments and traditional toy play, and asked whether these other factors explained any individual differences in the use of ISL across the two contexts.

## 4 AIMS OF THE STUDY

In middle childhood, children engage with and enjoy play with toy figures and video games (Case‐Smith & Kuhaneck, 2008; Ofcom, 2019). Although it is well‐established that free play is a rich context for ISL in middle childhood (Paine et al., 2019a), the nature of children's speech as they engage with video games has received little attention. As such, in the context of a prospective longitudinal study, we investigated seven‐year‐olds' propensity to refer to internal states during solitary play with toy figures and as they played a video game. We aimed to: (1) establish whether children used ISL in both types of play; (2) examine the total use of ISL in the two contexts; (3) explore the different categories and referents of ISL in children's speech in the two types of play for descriptive purposes; and (4) investigate whether children's overall propensity to use ISL was consistent across both types of play while controlling for factors known to be associated with social understanding, including working memory (Bock et al., 2015; Carlson & Moses, 2001; Devine & Hughes, 2014), verbal ability (de Rosnay & Hughes, 2006; Slade & Ruffman, 2005), risk for socioeconomic disadvantage (Cole & Mitchell, 2000), and their mothers' propensity to refer to internal states (Ensor et al., 2014). Based on the literature reviewed, we hypothesized that there would be some consistency in the use of ISL between the two contexts (Carr et al., 2018; Longobardi et al., 2014), but we also expected subtle differences to be present in the use of ISL due to the different properties of the two contexts (Howe et al., 2014; Howe & Bruno, 2010; Trawick‐Smith et al., 2010).

## 5 METHOD

### 5.1 Design

The Cardiff Child Development Study (CCDS) is a prospective longitudinal study of a nationally representative sample of first‐time parents and their children. Three hundred and thirty‐two primiparous women and their partners (86% biological fathers) were recruited between 1 November 2005 and 31 July 2007 from National Health Service (NHS) prenatal clinics in hospitals and general practice clinics in Cardiff and The Vale University Health Board and the Gwent Healthcare Trust, United Kingdom. Midwifery teams also granted the recruitment team access to specialist prenatal clinics for medical problems and outreach services for vulnerably housed pregnant women, which enhanced the representativeness of the sample. No exclusion criteria were set, either for the recruitment during the pregnancy or after the baby was born, except in the case of miscarriage, the infant's death, or the infant's experience of health problems that were so severe, it would not be possible to participate in the study. Translators were employed for families whose native language was not English or Welsh, or for participants who had impaired hearing. Data collection took place in pregnancy (Wave 1) and at a mean of 6, 12, 21, and 33 months postpartum (Waves 2–5, respectively). The final assessment (Wave 6) is the focus of the present paper, and took place when the children were between 6.5 and 7.5 years of age (mean 83 months). The sociodemographic characteristics of the sample recruited into the CCDS are displayed in Table 1. The CCDS was found to be nationally representative when compared to a subsample of firstborn children in the Millennium Cohort Study, the most recent national birth cohort study in the United Kingdom (Kiernan, personal communication, 2009).

**TABLE 1:** Sociodemographic characteristics of the sample recruited (*N* = 332) into the Cardiff Child Development Study (CCDS)

Age at first birth (mean)	
Mother	28.15 (SD 6.35, range 16.09–42.99)
Father	30.81 (SD 6.82, range 15.62–30.81)
Social class (%)	
Middle class	50.9
Working class	49.1
Mother's education (%)	
No qualifications	5.1
Less and 5 GCSEs A* = C/basic, for example, key skills, NVQ, NNEB	16.6
5+ GCSEs A*‐C or GNVQ higher level	13.9
A‐levels A*‐E/BTEC/HNC	11.7
Undergraduate degree (BA or BSc)/HND	28.0
Postgraduate degree, for example, MSc, MD, PhD, PG Cert	24.7
Relationship status at the child's birth (%)	
Married	50.3
Cohabiting	33.7
In a relationship but not living together	6.3
Single	9.6
Ethnicity (%)	
British	92.7
Non‐British	7.3

### 5.2 Participants

The middle childhood assessment (Wave 6) took place at a target age of 7 years (*M* = 6.96, *SD* = 0.38). Of the original 332 families recruited, 22 families had withdrawn from the study and one family had never been traced, leaving 309 (93% of those recruited) remaining in the sample. Of those, 287 (93%) provided data at the middle childhood assessment, 272 being seen in the home and 15 only completing questionnaires. The present analyses focus on 251 children (92% of those seen in the home; *M* = 6.95 years, *SD* = 0.38) who had completed a free play activity with Playmobil figures and played a novel video game designed for the study; 111 girls (44%) and 140 boys (56%) did so (this gender composition reflects the fact that more boys [57%] than girls [43%] are in the full [*N* = 332] CCDS sample). Six children refused to complete at least one of the activities: one did not play the game due to time restrictions; one did not complete any child testing; and one could not be assessed on these tasks due to a severe developmental delay. One family withdrew their data after the assessment and one session took place in a language other than English or Welsh and no translation was available. In 10 cases, technical problems resulted in data being unavailable for at least one of the tasks. The 251 children in the present sample were not significantly different from the original *N* = 332 with respect to sociodemographic adversity scores, (*p* > .05).

### 5.3 Procedure

At the middle childhood assessment, families were visited at home for two 2‐hr assessment sessions. The primary caregiver (97% mothers) completed interviews with one researcher while the firstborn child completed a battery of cognitive, social, and emotional tasks with a second researcher. These tasks included a battery of age‐appropriate social understanding tasks that used Playmobil figures: second‐order false belief, social information processing, Machiavellian intelligence, and simple deception tasks (Paine et al., 2018; Christie & Geis, 1970; Leekam & Prior, 1994; Quiggle, Garber, Panak, & Dodge, 1992). These tasks were followed by a free play activity with Playmobil figures and then the video game, which were the focus of the present study. If necessary, a third researcher kept any younger siblings occupied to prevent disruption of the interviews or assessments. At the end of the final session, the focal child, caregiver, and any siblings present then took part in a series of interaction tasks. At the end of the session, the child was given a £10 book voucher, and the caregiver was given a £20 gift voucher.

### 5.4 Materials

**FIGURE 1:**
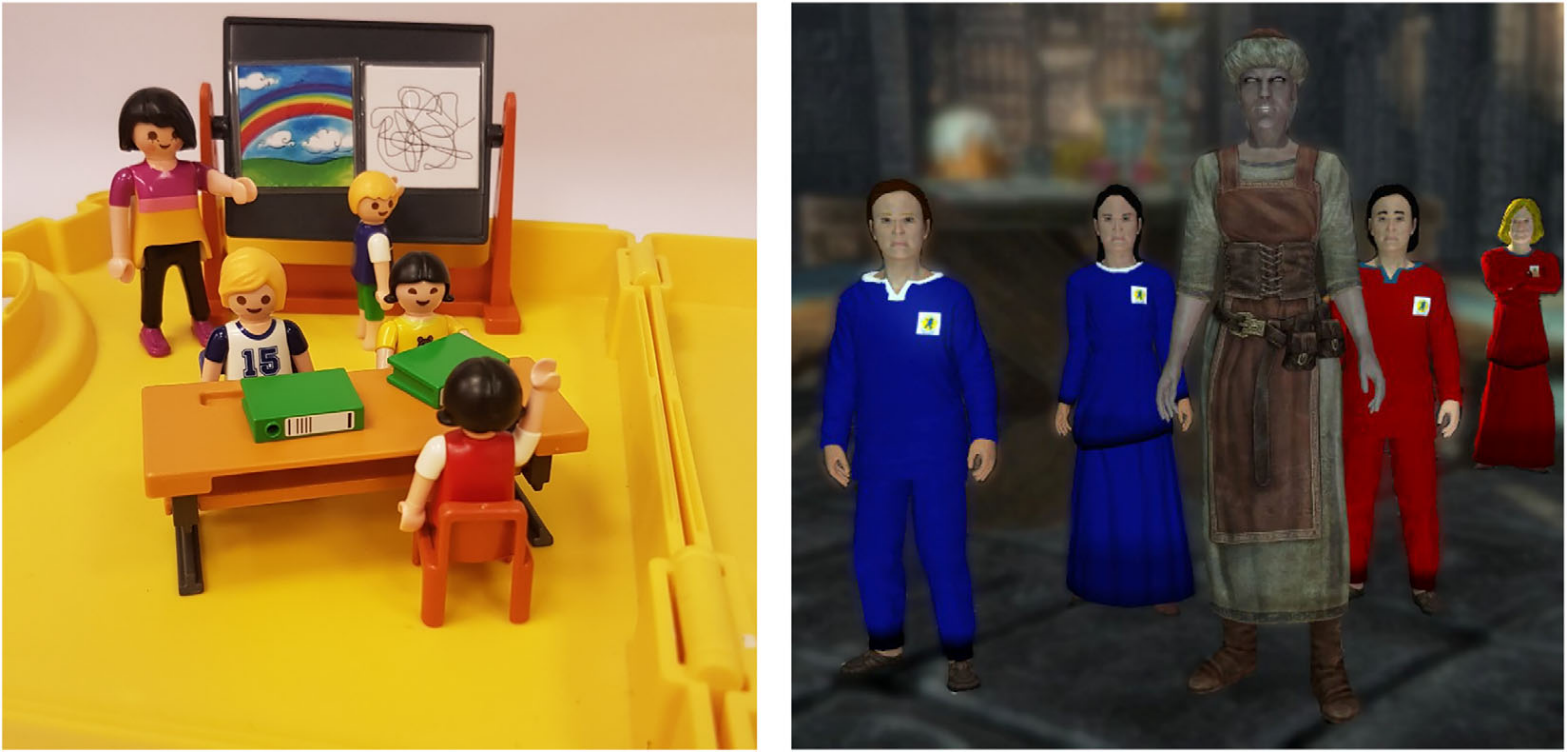
A selection of the characters that appear in the two contexts of play. The image on the left depicts the children and teacher from the Playmobil activity, and the image on the right depicts the children and Storyteller from the CAMGame

*Free play with Playmobil figures*. In the first home visit, after completing the social understanding tasks, the children were given an opportunity to play with Playmobil figures in any way they would like on their own for 3 minutes (see Figure 1). Experimenters were encouraged to engage with the children's play only at the child's request (see Paine et al., 2019a, for more details). Available Playmobil included all sets and figures that had been used in the preceding social understanding battery; a bedroom, laundry, and school set, with four children and two adult figures. Children freely played with the Playmobil toys for an average time of 2 min and 52 s (*SD* = 22 s, *range* = 45 s–3 min).

*The Castell Arth Mawr Adventure Game*. The Castell Arth Mawr Adventure Game (CAMGame; Hay et al., 2017) is a first‐person perspective game that was modified from the commercially available game The Elder Scrolls V: Skyrim (Bethesda, 2011) using freely available modification tools. The commercial game was modified to create a narrative that consisted of 11 “scenes” portraying the child on a school trip to a castle with their teacher and classmates, identified by the red school sweatshirts they wore, and encountering children wearing blue sweatshirts from a rival school (see Figure 1). This modification allowed us to present children with the same series of emotional challenges that might provoke prosocial behaviour, fear‐related behaviours, or aggressive responses using a mallet that their character had been given at the start of the game (for further details of these specific challenges, see supplementary materials for a detailed narrative of the game. A video demonstration of the CAMGame can be found at https://youtu.be/SpixvsHypg8).

Children also completed the CAMGame during the first middle childhood home visit. Children were told that in the game they would take part in a school trip to the castle; they had to stop and listen to the characters in the game to find out where to go and what to do. The children's speech and faces were video‐recorded using the webcam on an Alienware™ laptop using an Xbox™ controller with the right trigger coloured in purple and the left analogue stick coloured white to correspond with instructions given by the game. The researcher explained how to use the controller before the child began playing the game, with reminders given as a part of the narrative. Children varied considerably in how long they took to complete the game, taking on average 19 min and 5 s (*SD* = 5 min and 45 s, *range* = 8 min and 30 s–41 min and 45 s).

### 5.5 Measures

*Children*'*s talkativeness*. Video recordings of children's speech while playing with the Playmobil figures and the CAMGame were transcribed into 5‐sec segments. Trained translators transcribed tasks that took place in Welsh. Time segments in which children were not playing the game due to a technical error, were repeating a part of the game that they had already completed, or were not engaged in either of the tasks, were excluded from all analyses, including calculations of talkativeness scores and task length. A proportional measure of the child's talkativeness was generated by dividing the number of 5‐sec segments in which the child spoke by the total number of 5‐sec segments of the length of the task, yielding a score between 0 and 1. Any instances of non‐word vocalizations that were not sound effects were excluded from these calculations. This time sampling measure of talkativeness has been validated as a measure of how much someone talks using *Audacity* voice analysis software on a subsample of video records, which yielded a measure of the precise duration of speech (Roberts et al., 2013).

*Children*'*s references to internal states*. The transcripts of children's speech as they played with the Playmobil and played the CAMGame were coded for the use of ISL using a coding scheme previously designed for the study (Paine et al., 2019a). This coding scheme was derived from Roberts and colleagues'  (2013) scheme, which was based on Bartsch and Wellman's (1995) belief‐desire categorization of the theory of mind. Each 5‐sec segment was considered in terms of whether it contained an utterance that included the use of ISL. The coding scheme categorizes speech that includes references to the following internal state categories: *perception*, *physiology*, *preference*, *intention*, *desire*, *emotion*, and *cognition*. The referent of each use of ISL was coded as referring to an inner state of the self, the characters (game characters or Playmobil figures), or others (e.g., the experimenter) (See Table 2 for descriptions and verbatim examples of each category for each referent). For the CAMGame, an additional referent category was included to capture attributions about the inner states of the virtual avatar that children controlled. Such instances of ISL were coded conservatively and recorded only when a term was used in the first person (plural or singular) but could not reflect the child's internal state in reality (e.g., “Oh no, I'm dying!”). In any cases where the referent of the term was unclear, it was coded as being attributed to “other” individuals.

**TABLE 2:** Internal state language (ISL) coding scheme developed by Paine et al., (2019a) with verbatim examples from the Playmobil free play activity and the CAMGame

ISL category	ISL category description	Examples from Playmobil activity			Examples from CAMGame		
		ISL to self	ISL to character	ISL to other	ISL to self	ISL to character	ISL to other
Perception	Comments made about the perception of an object using one of five senses, such as “see,” “hear,” “feel,” “taste,” “smell.”	“*That one looks like…*”; “*I didn*'*t see it*”	“*She heard it stop again*”; “*And the teacher sees*”	“*Can you see it in the camera?*”	“*I*'*m looking at the floor*”	“*What are you looking at me for?*”	“*Did you see that guy?*”
Physiology	Comments made about physical states and sensations, including “sleepy,” “pain,” “hot/cold (as in temperature),” “sick,” “comfy.”	“*It really hurts*”	“*They*'*re feeling tired*”	No instances of physiology to other occurred	“*Ooh I*'*ve got pins and needles*”	“*Am I actually hurting my own people?*”	“*Did you die in this one*”
Preference	Comments made about positive or negative judgements of an object, action or experience. Coding preference includes terms include “like,” “hate,” “love,” “favourite,” “enjoy,” “interest.”	“*My favourite colour is pink and blue*”; “*I like this*”	In play voice: “*Kate*'*s the best*”	“*Do you like that thing that*'*s there?*”	“*I don*'*t like this game*”	“*Why do you not like me?*”	No instances of preference to other occurred
Intention	Comments made about present intentional actions that are goal‐directed and future intentions. Includes “try,” “attempt,” “on purpose,” “mean to,” “going to.”	“*I*'*m just gonna mix them all*”; “*I*'*m going to do this*”	In play voice: “*I*'*m gonna find a bed*”; “*They*'*re gonna sit down*”	“*Can you try and put them in?*”; “*Are you gonna play?*”	“*I*'*m going to go through the door*”	“*Are you not gonna push me?*”	“*He [dog in room] tries to get attention*
Desire	Comments made about longing for an object, action or experience. Desire terms include “want,” “wish,” “hope,” “fancy,” “rather,” “need (as in want).”	“*I wish you can buy everything for free*”; “*I want that one*”	In play voice: “*Actually I don*'*t want to…*”; “*And he wants to sit there*”	“*Do you wanna play with me?*”	“*I don*'*t wanna open it*”	“*Why does he want me to smash it?*”	“*You wanna eat it?*” *(In response to experimenter)*
Emotion	Comments made about feeling states, including basic emotions “happy,” “sad,” “surprised,” “disgusted” and variations like “fed up,” “bored,” “glad,” “excited.”	“*That was disgusting*”	“*Mum and teacher are happy*”; “*They are kind of sad*”	No instances of emotion to other occurred	“*I*'*m a bit scared*”	“*He*'*s scared, he*'*s a scaredy cat*”	“*Would you be scared if you was only a school person going here?*”
Cognition	Comments made about beliefs and knowledge. Also include general terms indicating other cognitive activity, such as “remember,” “imagine,” “pretend,” “understand.”	“*I*'*m gonna pretend these chairs are here*”; “*I think that goes here*”	“*Kate does not know where it is*”; “*Then they thought*”	“*Amd imagine she buyed another*”	“*I probably know already*”	“*Does he know where the magic statue is?*”	“*You thought I wouldn*'*t find the treasure*”

An independent observer coded the frequency of children's use of ISL for a random sub‐sample of 67 (26%) of the transcripts of children's play with the toy figures and 52 (20%) of the transcripts of children's speech during the CAMGame, median *ICC* = .95 for play with toy figures and .94 for the CAMGame. As the two play contexts varied in duration and the amount of speech varied between participants and contexts, proportion scores were created to better enable comparisons of children's use of ISL in each task, with the total frequency of each ISL category divided by the total number of 5‐sec segments for the task. Although previous research investigating ISL computes proportion scores by dividing the frequency of internal state references by the measure of talkativeness (e.g., Howe et al., 2014), we opted not to compute proportions in this way, because the amount of speech varied between participants and between contexts.

*Family adversity*. Sociodemographic measures characterizing the family environment were assessed from mothers' reports when the study began; maternal variables were used to ensure an identical source of information for each family, regardless of whether or not the child's father was present in the home and participating in the study. An index of children's risk for family adversity was created using polychoric principal components analysis (PCA). The mothers' experiences that contributed to this index were: (a) not having achieved the minimum level of qualifications required for the completion of secondary education in the United Kingdom (i.e., less than five exam passes or equivalent attainments); (b) being 19 years of age or under at the time of their child's birth; (c) not being legally married during pregnancy; (d) being in an occupation classified as lower status according to the Standard Occupational Classification 2000 (SOC2000; Elias, McKnight, & Kinshott, 1999), and (e) not being in a stable partnership with the firstborn child's father (i.e., married, cohabiting, or in a committed relationship without living together). All the items contributed to a single component which explained approximately 77% of the shared variance with positive scores indicating a higher than average level of adversity at the time of the child's birth.

*Children*'*s early conversational environment*. At the early infancy home assessment (Wave 2), mothers and the focal child (*M* age = 6.64 months, *SD* = 0.88) had been given a topic sharing task using an activity board: a plastic toy with folding flaps and images of cartoon animals. Mothers were asked to show the toy to the infant for 2 min, and this interaction was video recorded, transcribed, and coded for mothers' references to ISL using the same coding scheme described above (see Table 2; Paine et al., 2019a). Inter‐rater reliability was established between two independent coders on 65 (31.4%) of mothers' references to ISL in early infancy (median *ICC* = .99). Data were available for 222/251 (88%) families in the present sample. Fourteen families did not take part in this assessment, and data were not available for 15 of the families for this task.

*Caregivers*' *reports of children*'*s play with toy figures and video games*. As part of a general interview about the child during the middle childhood assessment, caregivers were asked to identify “what kind of things does [child's name] like to do?” from a list of 18 activities. Two variables were derived from the caregivers' reports: (1) a composite variable measuring liking to *play with toy figures* (including Playmobil figures, action figures, toy vehicles, and dolls) and (2) liking to *play video/computer games*. Both variables were dichotomous, where 0 indicated not liking to do the activity and 1 liking to do the activity. Data on these measures were available for all children.

*Verbal ability*. In session 1 of the middle childhood assessment, children's receptive vocabulary was assessed using the British Picture Vocabulary Scale (BPVS; Dunn & Dunn, 2009). BPVS data were available for 247 (98%) of the subsample for this paper: three children did not complete the task and one child was not testable.

*Working memory*. Also in session 1 of the middle childhood assessment, children completed the Visuo‐Spatial Sequencing (VSS) task from the Amsterdam Neuropsychological Tasks (ANT; de Sonneville, 1999). Children were presented with nine circles on the computer screen presented in a square matrix. Following a beep, an animated hand on the screen pointed to a sequence of the circles that gradually increased in the number of targets and the complexity of the sequence. The child was asked to replicate this sequence of circles; the total number of correctly identified targets in the correct order measured their working memory (Paine et al., 2019a). Data on this task were available for 237 children: two children refused to participate in the task and two were not testable; seven did not complete the task due to time constraints or incomplete visits; and, for three, data were unavailable due to technical errors.

### 5.6 Data analysis

We first describe features of children's speech in the two contexts of play in terms of the overall use of ISL, and the categories and referent of internal states. We have used data that reflect the proportion of the use of ISL based on the task length, not absolute frequencies, to account for differences in how long children played with the toys and the video game. However, descriptive data regarding absolute frequencies of the use of ISL are presented in Table 3. Because the data were not normally distributed, nonparametric analyses (Wilcoxon signed‐rank tests, Spearman's Rho) were used for analyses involving the ISL variables and child characteristics. Significant associations (*p* < .05) were carried forward and used as control variables for subsequent regression analyses. The proportion scores for children's ISL in the CAMGame were transformed for use as the outcome variable in the regression using the logit transformation function in R (R Core Team, 2018), as this is the preferred method of improving the normality of proportional data (Warton & Hui, 2011).

**TABLE 3:** Means and standard deviations (SD) of internal state language according to category and referent

	Playmobil free play		CAMGame	
	Frequencies (mean, [SD])	Proportions (mean, [SD])^a^	Frequencies (mean, [SD])	Proportions (mean, [SD])^a^
Total	3.42 (3.13)	0.10 (0.09)	19.41 (16.77)	0.08 (0.06)
Perception	0.44 (0.80)	0.01 (0.02)	4.07 (4.50)	0.02 (0.02)
Physiology	0.15 (0.50)	0.00 (0.01)	0.41 (0.99)	0.00 (0.00)
Preference	0.16 (0.54)	0.00 (0.02)	0.63 (1.16)	0.00 (0.01)
Intention	0.79 (1.53)	0.02 (0.05)	4.43 (4.98)	0.02 (0.02)
Desire	0.67 (1.12)	0.02 (0.03)	2.33 (3.28)	0.01 (0.01)
Emotion	0.08 (0.31)	0.00 (0.01)	0.78 (1.60)	0.00 (0.01)
Cognition	1.12 (1.42)	0.03 (0.04)	6.76 (7.45)	0.03 (0.02)
ISL attributed to self	1.71 (2.15)	0.05 (0.06)	16.50 (14.77)	0.07 (0.05)
ISL attributed to character	1.40 (1.87)	0.04 (0.05)	1.63 (2.32)	0.00 (0.00)
ISL attributed to other	0.31 (0.78)	0.01 (0.02)	1.13 (1.81)	0.00 (0.01)
ISL attributed to avatar	N/A^b^	N/A^b^	0.14 (0.55)	0.00 (0.00)

Abbreviation: ISL, internal state language.

^a^ Means and standard deviations for ISL variables as proportions are based on task length.

^b^ No descriptive statistics are reported in relation to the avatar for the Playmobil free play activity, as this referent was only coded in the CAMGame.

## 6 RESULTS

### 6.1 Children's speech during play with toys and the video game

The descriptive statistics for children's speech in each context are presented in Tables 3 and 4. All speech variables entered into analyses are as a proportion of task length. On average, children spoke for a greater proportion of time when playing with Playmobil Figures (*M* = .62, 95%, *SD* = .62) than during CAMGame (*M* = .45, *SD* = .19), *z* = 9.46, *p* < .001.

**TABLE 4:** Means, standard deviations, and intercorrelations of overall internal state language variables and relevant child characteristics

	1	2	3	4	5	6	7	8	9	10
1. Playmobil total ISL	—									
2. Playmobil talkativeness	.59**	—								
3. CAMGame total ISL	.31**	.39**	—							
4. CAMGame talkativeness	.28**	.46**	.79**	—						
5. Age	.05	−.02	.02	.00	—					
6. Gender	−.03	.07	.03	.05	.03	—				
7. Verbal ability	.01	.06	.10	.04	−.21**	−.08	—			
8. Working memory	−.07	−.06	−.17*	−.18**	.22**	−.14*	.26**	—		
9. Sociodemographic adversity	−.03	−.02	.08	.15*	.18**	.11	−.40**	−.23**	—	
10. Mothers' use of ISL in early infancy	−.04	−.04	−.02	−.06a	−.24**	.05	.22**	.08	−.27**	—
*Mean*	.10	.62	.08	.45	83.45	1.56	99.26	66.19	−.08	1.80
*SD*	.09	.26	.06	.19	4.53	.50	11.78	18.39	.96	1.55

Abbreviations: CAMGame, Castell Arth Mawr Adventure Game; ISL, internal state language.

*Note*: * *p* < .05; ** *p* < .01. Means and standard deviations for ISL variables are based on proportions of task length. All correlations are Spearman's rho.

When playing with the Playmobil, 209 children (83.27%) referred to at least one internal state, whereas while playing the video game, 248 children (98.80%) made a reference to at least one internal state. However, children referred to more internal states when playing with Playmobil Figures (*M* = .10, *SD* = .09) than during CAMGame (*M* = .08, *SD* = .06), *z* = 2.07, *p* = .04.

### 6.2 Children's use of ISL in play with toys and the video game

**FIGURE 2:**
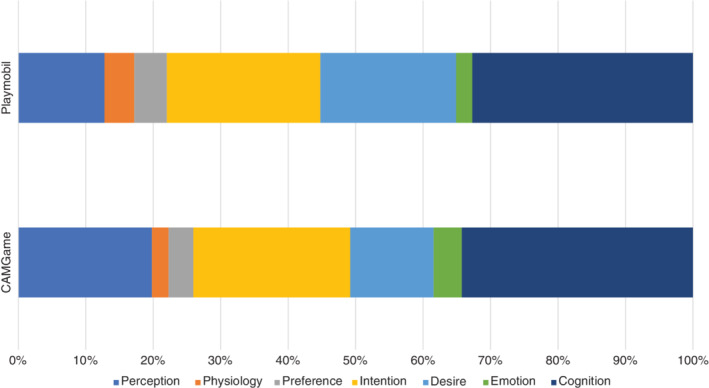
Relative proportion of the use of internal state language categories in the two contexts of play

*ISL categories*. Figure 2 displays the relative proportions of ISL categories in the two play contexts; descriptive statistics for the use of ISL according to the category are presented in Table 3. When playing with Playmobil and when playing the CAMGame, children referred to cognitions more than any other internal state category (*M* = .03, *SD* = 0.04 for the Playmobil free play activity; and *M* = .03, *SD* = 0.02 for the CAMGame). The descriptive statistics presented in Figure 2 and Table 3 indicate subtle differences in the number of references to ISL categories between the two contexts; however, due to the low proportion scores in certain ISL categories, further analyses were not conducted.

*The referent of children*'*s ISL*. Table 3 presents the descriptive statistics for the referents of children's ISL. Children referred to their own internal states the most in the two contexts of play (*M* = .05, *SD* = 0.06 for the Playmobil free play activity; and *M* = .07, *SD* = 0.05 for the CAMGame). However, children referred to their own internal states more often when playing the CAMGame (*M* = .069, *SD* = .051) than when playing with the Playmobil toys (*M* = .051, *SD* = .063), *z* = 4.95, *p* < .001, and referred to the internal states of characters more when playing with the Playmobil (*M* = .040, *SD* = .055) than during the CAMGame (*M* = .007, *SD* = .010), *z* = 9.11, *p* < .001.

### 6.3 Correlates of children's use of ISL

Table 4 presents the means, standard deviations, and intercorrelations between the total use of ISL (as a proportion of the length of the activity) in each context and child and family characteristics. Two hundred and six (82.1%) of the children were reported as liking to play with toy figures, and 220 (87.6%) were reported as liking to play with video/computer games, confirming that the two types of play studied were relatively popular in this sample. As these variables were at ceiling, they were not included in the present analyses. Children's use of ISL was not significantly associated with family adversity or the early conversational environment. Nor was ISL associated with the child's age, gender, or verbal ability. Children's working memory scores were associated with their references to internal states during the CAMGame, but working memory was not correlated with ISL during play with the toy figures.

### 6.4 Consistency in the use of ISL in play with toys and the video game

Children's total references to internal states when playing with toys and while playing the CAMGame were positively correlated. Children's overall talkativeness was consistent across contexts, and was positively associated with ISL in both contexts (all *p*s < .05, see Table 4). Therefore, a mean talkativeness score was computed to indicate children's propensity to talk which was used in subsequent analyses.

To investigate whether children's overall propensity to use ISL was consistent across both types of play, we entered children's ISL during free play as a predictor in a regression analysis, with children's ISL in the CAMGame as the outcome variable; covariates of children's ISL (mean talkativeness and working memory) were controlled. When these variables were entered into the analysis, only children's talkativeness (*β* = .72, *p* < .001, 95% CI = [1.99, 2.70]) significantly predicted children's ISL in the CAMGame in the final model (see Table 5). Children's use of ISL while playing with Playmobil figures did not account for significantly more variance than the child factors alone, as it did not represent a significant step in the model, *F*(1, 229) = .07, *p* = .067, *ΔR* ^*2*^ = .01.

**TABLE 5:** Prediction of the use of internal state language in the CAMGame from use of internal state language during the free play activity, controlling for covariates

Predictor	Δ*R* ^*2*^	*B*	*SE B*	*β*	95% CI
Step 1	.46**				
Constant		−3.40	.15		−3.69 to −3.11
Working memory		−0.00	.00	−.04	−0.01 to 0.00
Mean talkativeness		2.18	.16	.67**	1.87 to 2.49
Step 2	.01				
Constant		−3.41	.15		−3.70 to −3.12
Working memory		−0.00	.00	−.04	−0.01 to 0.00
Mean talkativeness		2.34	.18	.72**	1.99 to 2.70
Overall ISL use in free play		−0.75	.41	−.10	−1.56 to 0.54

*Note*: * *p* < .05; ** *p* < .01. The coefficients presented are those obtained in the final model: *F*(3, 233) = 67.18, *p* < .001, *R* ^*2*^ = .46.

Abbreviation: ISL, internal state language.

With respect to the categories of ISL, children's references to *preferences*, *r*_*s*_(251) = .17, *p* = .008, *intentions*, *r*_*s*_(251) = .15, *p* = .018, and *cognitions*, *r*_*s*_(251) = .20, *p* = .002, were positively correlated across the two types of play. With respect to the referent of the internal state attribution, children's references to their own, *r*_*s*_(251) = .26, *p* < .001, the characters', *r*_*s*_(251) = .18, *p* = .005, and other people's, *r*_*s*_(251) = .14, *p* = .033, internal states were positively associated across the two contexts. Because the association between children's total use of ISL in the two contexts was explained by children's talkativeness scores, these univariate analyses were not explored further.

## 7 DISCUSSION

### 7.1 Summary of the findings

Children used ISL when playing a video game and when they played with toy figures. Most children referred to at least one internal state while playing the video game, though they referred to fewer internal states than when playing with Playmobil figures. The relationship between children's ISL in the two contexts of play was explained by children's general talkativeness. Our findings support previous research indicating that children's free play is a rich context for ISL (Leach et al., 2017) and extend this to include play in virtual environments. Our findings corroborate earlier qualitative research demonstrating that children narrate their video game play (Søndergaard, 2013), and show they refer to the inner worlds of purely virtual characters when doing so. Our findings support earlier claims that video games can be considered a form of play that children engage with in a similar way to more traditional play with toys (Lillard, 2014; Singer & Singer, 2005).

### 7.2 Children's use of ISL when playing with toy figures and video games

Children referred to more internal states when playing with Playmobil figures than the video game, and there were also differences in how ISL was used in each context. In line with research on mothers' use of ISL in different contexts (Carr et al., 2018; Howe, Rinaldi, & Recchia, 2010), and studies of other elements of children's speech (Paine et al., 2019b; Krafft & Berk, 1998), our findings indicate that differences in the structure and presentation of play activities afford different opportunities to discuss internal states. Children referred to *characters*' internal states more often when playing with toy figures, and referred to their *own* internal states more often during the video game. The open‐ended format of the play with toys afforded the children the opportunity to engage in different types of play, including telling stories that mentioned characters' internal states (e.g., “*They want* to visit the animals”; “*Alex wants* the ball”). They might also enact character roles using the figures (e.g., “[In play voice] I *hate* bedtime”).

Our findings indicate that there also may be differences in the categories of ISL used in the different contexts of play. The unscripted free play session may provide children more opportunities to discuss their own *desires*, particularly in terms of the way in which they played and preferred to set up the toys (e.g., “I *wanna* put these on the washing line”). In contrast, when playing the video game, children were restricted in their possible actions by the constraints and rules of the game (Valkenburg, 2001), and so may have been less likely to express desires that could not be met. However, children may have referred to their *emotions* and *perceptions* more often when playing the video game than when using the Playmobil figures for several reasons: because the presentation of the game was perceptually engaging (e.g., “I *hear* someone from the blue school”); because some of the challenges built into the game involved searching for items (e.g., “I *see* the magic statue!”); or because the graphics and narrative of the game elicited emotional responses (e.g., “I'm a bit *scared* what's come, cause I've got loads of monsters in here^1^”).

The pre‐scripted nature of the virtual characters in the video game may have limited opportunities for children to speak about characters' internal states; however, the first‐person perspective and challenging nature of CAMGame may have promoted private speech (Fernyhough & Fradley, 2005), particularly in the children's commentary on their ongoing activity (e.g., “*I think I see* something”; “*I think I*'*m just gonna* go”). Given the story‐based nature of the game, much of this speech may also reflect children's interactions with the characters (e.g., “Blue boys they're stupid, stupid, *I*'*m going to* get ready to hit you”; “Where do *you think* you're going? *I*'*m scared*.”). Evidence from studies exploring associations between stories and social cognition (both from storybooks and other narrative media; Mar, Tackett, & Moore, 2010; Mar, 2018) suggests that fictional narratives afford opportunities to “abstract and simulate the social world” (Mar & Oatley, 2008, p. 185). Indeed, evidence suggests that in‐game storytelling is positively associated with social understanding; this association, it is suggested may occur via players' opportunities to engage in virtual social interaction (Bormann & Greitemeyer, 2015; Mar, 2018). These processes and potential links with social understanding, however, require further investigation.

It is notable that, when coding children's speech while playing the video game, we used a conservative method to distinguish between the attributions of internal states to the self versus the avatar controlled by the child. We could only reliably code references to one internal state for the avatar, the avatar's physiology (e.g., “That might *hurt* me”), as this was the only category of ISL in which there was no possibility that the child meant to attribute the internal state to the self. Such speech could be interpreted as children integrating themselves with the avatar, resulting in the referent being difficult to separate in their use of language (Klimmt et al., 2009). Therefore, it is possible that some of the references made to children's own internal states were in fact attributions to the avatar.

### 7.3 Limitations of the study

Our study has limitations. The experimenter's presence during both forms of play could have affected children's use of ISL. However, the evidence is mixed regarding whether the presence of adults leads to differences in the frequency and quality of children's speech during play, depending on their level of involvement (Howe & Bruno, 2010; Krafft & Berk, 1998). In our study, experimenters were advised to engage with the play only at the child's request; although this potentially may have differed across experimenters, our analyses indicated no differences in different experimenters' speech or use of ISL. However, future research could make use of unobtrusive data recording methods to record children's speech during their play in the absence of an experimenter or observer.

Furthermore, we only investigated children's use of ISL during solitary play. ISL is most often studied in the context of social interaction. Solitary imaginative play activities have been argued to be social in that they are performances to real, or imagined, others (Piaget, 1962). In the present study, the experimenter's presence may have provided an audience for the child's performance. Additionally, our findings corroborate previous work on a solitary play that found similar qualities in children's private speech when playing alone (Davis et al., 2013; Krafft & Berk, 1998). Our work extends these findings to include both playing with toys and with video games. Future research could investigate children's use of ISL when engaging in and negotiating to play video games with a peer, as opposed to more commonly studied social play with toys (Howe et al., 2014; Leach et al., 2015).

Finally, order effects may have contributed to the differences found in relation to the children's use of ISL in the Playmobil free play activity and when playing the video game. The social understanding tasks, the free play activity, and the CAMGame were presented in a fixed order within the task battery, and it is possible that the stories told in the social understanding tasks may have primed patterns of ISL in the free play activity, which further prompted patterns of ISL present in the CAMGame. Although counter‐balancing the order of the presentations of these tasks would have been ideal, these activities formed part of a larger battery of tasks, including the covariates included in the present analyses, for the time‐intensive home visit. The order of tasks was devised to ensure data were collected efficiently, and children's interest was sustained throughout the duration of the visit. However, the possibility that order effects might have had an effect calls for caution to be taken in the interpretation of the present findings and warrants future research that can address this issue.

## 8 CONCLUSIONS

In summary, we have demonstrated that children's video game play can be used as a new context for the study of children's references to internal states. Children used ISL in both virtual and traditional play contexts, but their speech about internal states in the two contexts differed in terms of the nature and referent of the inner states. Our results have implications for parents, teachers, and researchers seeking to foster children's developing social understanding by encouraging conversations about mental states (Bianco, Lecce, & Banerjee, 2016). We have found that children do indeed refer to internal states when playing video games, and therefore this popular activity could be targeted to support children's social understanding by promoting their use of ISL. At a time when concerns are being expressed by parents and policymakers about children's screen‐based activities, our findings demonstrate that children demonstrate their social understanding and imaginative skills when playing video games, just as they do when they are engaged in more traditional forms of play with toys.

## CONFLICT OF INTEREST

The authors of this manuscript have no conflicts of interest to declare.

## Supporting information

supplementary materials

## Data Availability

The data that support the findings of this study are available on request from the corresponding author. The data are not publicly available due to privacy or ethical restrictions.

## Endnote

^1^
No monsters were present in the game.

## References

[icd2223-bib-0001] Anderson, C. A., & Bushman, B. J. (2018). Media violence and the general agression model. Journal of Social Issues, 74(2), 386–413. 10.1111/josi.12275

[icd2223-bib-0007] Bartsch, L., & Wellman, H. M. (1995). Children talk about the mind. New York, NY: Oxford University Press.

[icd2223-bib-0008] Bethesda . (2011). The elder scrolls V: Skyrim. Rockville, MD: Bethesda Softworks.

[icd2223-bib-0009] Bianco, F., Lecce, S., & Banerjee, R. (2016). Conversations about mental states and theory of mind development during middle childhood: A training study. Journal of Experimental Child Psychology, 149, 41–61. 10.1016/j.jecp.2015.11.006 26723472

[icd2223-bib-0010] Bock, A. M., Gallaway, K. C., & Hund, A. M. (2015). Specifying links between executive functioning and theory of mind during middle childhood: Cognitive flexibility predicts social understanding. Journal of Cognition and Development, 16, 509–521. 10.1080/15248372.2014.888350

[icd2223-bib-0011] Bormann, D., & Greitemeyer, T. (2015). Immersed in virtual worlds and minds: Effects of in‐game storytelling on immersion, need satisfaction, and affective theory of mind. Social Psychological and Personality Science, 6, 646–652. 10.1177/1948550615578177

[icd2223-bib-0012] Brown, E., & Cairns, P. (2004). A grounded investigation of game immersion. Paper presented at CHI'04. Conference on Human Factors in Computing Systems. Vienna: Austria, 1297–1300.

[icd2223-bib-0013] Cairns, P., Cox, A., & Nordin, I. A. (2014). Immersion in digital games: Review of gaming experience research. In M. C. Angelides & H. Agius (Eds.), Handbook of digital games (pp. 339–361). Hoboken, NJ: Wiley‐Blackwell.

[icd2223-bib-0014] Carlson, S. M., & Moses, L. J. (2001). Individual differences in inhibitory control and children's theory of mind. Child Development, 72, 1032–1053. 10.1111/1467-8624.00333 11480933

[icd2223-bib-0015] Carpendale, J. I., & Lewis, C. (2015). The development of social understanding. In R. Lerner (Ed.), Handbook of child psychology and developmental science (Vol. 2: Cognitive processes, pp. 381–424). New York, NY: Wiley Blackwell 10, 9781118963418.

[icd2223-bib-0016] Carr, S., Slade, L., Yuill, N., Sullivan, S., & Ruffman, T. (2018). Minding the children: A longitudinal study of mental state talk, theory of mind and behavioural adjustment from age 3 to age 10. Social Development, 27(4), 826–840. 10.1111/sode.12315

[icd2223-bib-0017] Case‐Smith, J., & Kuhaneck, H. M. (2008). Play preferences of typically developing children and children with developmental delays between ages 3 and 7 years. OTJR: Occupation, Participation and Health, 28(1), 19–29.

[icd2223-bib-0018] Christie, R., & Geis, F. (1970). Studies in Machiavellianism. New York, NY: Academic Press.

[icd2223-bib-0019] Cole, K., & Mitchell, P. (2000). Siblings in the development of executive control and a theory of mind. British Journal of Developmental Psychology, 18(2), 279–295. 10.1348/026151000165698

[icd2223-bib-0020] Copenhaver, A., Mitrofan, O., & Ferguson, C. J. (2017). For video games, bad news is good news: News reporting of violent video game studies. Cyberpsychology, Behavior and Social Networking, 20(12), 735–739. 10.1089/cyber.2017.0364 29148827

[icd2223-bib-0021] Davis, P. E., Meins, E., & Fernyhough, C. (2013). Individual differences in children's private speech: The role of imaginary companions. Journal of Experimental Child Psychology, 116, 561–571. 10.1016/j.jecp.2013.06.010 23978382 PMC3870270

[icd2223-bib-0022] Davis, P. E., Meins, E., & Fernyhough, C. (2014). Children with imaginary companions focus on mental characteristics when describing their real friends. Infant and Child Development, 23, 622–633. 10.1002/icd.1869 25685093 PMC4321191

[icd2223-bib-0023] de Rosnay, M., & Hughes, C. (2006). Conversation and theory of mind: Do children talk their way to socio‐cognitive understanding? British Journal of Developmental Psychology, 24(1), 7–37. 10.1348/026151005X82901

[icd2223-bib-0024] de Sonneville, L. M. J. (1999). Amsterdam neuropsychological tasks: A computer‐aided assessment program. In B. P. L. M. den Brinker, P. J. Beek, A. N. Brand, F. J. Maarse, & L. J. M. Mulder (Eds.), Cognitive ergonomics, clinical assessment and computer‐assisted learning (pp. 187–203). Lisse, The Netherlands: Swets and Zeitlinger.

[icd2223-bib-0025] Devine, R. T., & Hughes, C. (2014). Relations between false belief understanding and executive function in early childhood: A meta‐analysis. Child Development, 85(5), 1777–1794. 10.1111/cdev.12237 24605760

[icd2223-bib-0026] Downey, S., Hayes, N., & O'Neill, B. (2007). Play and technology for children aged 4–12. Centre for Social and Educational Research. Dublin Institute of Technology. Office of the Minister for Children.

[icd2223-bib-0027] Drummond, A., Sauer, J. D., & Ferguson, C. J. (2020). Do longitudinal studies support long‐term relationships between aggressive game play and youth aggressive behaviour? A meta‐analytic examination. Royal Society Open Science, 7(7), 200373. 10.1098/rsos.200373 32874632 PMC7428266

[icd2223-bib-0028] Dunn, L. M., & Dunn, D. M. (2009). The British picture vocabulary scale. London: GL Assessment Limited.

[icd2223-bib-0029] Elias, P., McKnight, A., & Kinshott, G. (1999). SOC2000: Redefining skill: Revision of the standard classification system. Skills Task Force Research Paper, 19.

[icd2223-bib-0030] Ensor, R., Devine, R. T., Marks, A., & Hughes, C. (2014). Mothers' cognitive references to 2‐year‐olds predict theory of mind at ages 6 and 10. Child Development, 85(3), 1222–1235. 10.1111/cdev.12186 24320094

[icd2223-bib-0031] Fernyhough, C., & Fradley, E. (2005). Private speech on an executive task: Relations with task difficulty and task performance. Cognitive Development, 20(1), 103–120. 10.1016/j.cogdev.2004.11.002

[icd2223-bib-0032] Francis, A., & Mishra, P. (2009). Is AIBO real? Understanding children's beliefs about and behavioural interactions with anthropomorphic toys. Journal of Interactive Learning Research, 20(4), 405–422.

[icd2223-bib-0033] Giffin, H. (1984). The coordination of meaning in the creation of a shared make‐believe reality. In I. Bretherton (Ed.), Symbolic play: The development of social understanding (pp. 73–100). Orlando, FL: Academic Press, Inc..

[icd2223-bib-0034] Goldman, A. (2006). Imagination and simulation in audience responses to fiction. In S. Nichols (Ed.), The architecture of the imagination: New essays on pretence, possibility, and fiction. New York: Oxford University Press.

[icd2223-bib-0035] Halbrook, Y. J., O'Donnell, A. T., & Msetfi, R. M. (2019). When and how video games can be good: A review of the positive effects of video games on well‐being. Perspectives on Psychological Science, 16(6), 1096–1104. 10.1177/1745691619863807 31672105

[icd2223-bib-0036] Harrington, B., & O'Connell, M. (2016). Video games as virtual teachers: Prosocial video game use by children and adolescents from different socioeconomic groups is associated with increased empathy and prosocial behaviour. Computers in Human Behavior, 63, 650–658. 10.1016/j.chb.2016.05.062

[icd2223-bib-0037] Harris, P. L. (2000). The work of the imagination (1st ed.). Oxford, England: Blackwell.

[icd2223-bib-0003] Hay, D. F., Johansen, M. K., Daly, P., Hashmi, S., Robinson, C., Collishar, S., & van Goozen, S . (2018). Seven‐year‐olds’ aggressive choices in a computer game can be predicted in infancy. Developmental Science, 21(3), e12576. 10.1111/desc.12576 PMC5947600 28736940

[icd2223-bib-0038] Howe, N. (1991). Sibling‐directed internal state language, perspective taking, and affective behaviour. Child Development, 62(6), 1503–1512. 10.1111/j.1467-8624.1991.tb01621.x 1786731

[icd2223-bib-0039] Howe, N., Abuhatoum, S., & Chang‐Kredl, S. (2014). “Everything's upside down. We'll call it upside down valley!”: Siblings' creative play themes, object use, and language during pretend play. Early Education and Development, 25, 381–398. 10.1080/10409289.2013.773254

[icd2223-bib-0040] Howe, N., & Bruno, A. (2010). Sibling pretend play in early and middle childhood: The role of creativity and maternal context. Early Education and Development, 21(6), 940–962. 10.1080/10409280903440638

[icd2223-bib-0041] Howe, N., Rinaldi, C. M., & Recchia, H. E. (2010). Patterns in mother‐child internal state discourse across four contexts. Merill‐Palmer Quarterly, 56(1), 1–20.

[icd2223-bib-0042] Hughes, C., & Dunn, J. (1998). Understanding mind and emotion: Longitudinal associations with mental‐state talk between young friends. Developmental Psychology, 34(5), 1026–1037. 10.1037/0012-1649.34.5.1026 9779748

[icd2223-bib-0043] Kane, S. R., & Furth, H. G. (1993). Children constructing social reality: A frame analysis of social pretend play. Human Development, 36, 199–214. 10.1159/000278207

[icd2223-bib-0044] Klimmt, C., Hefner, D., & Vorderer, P. (2009). The video game experience as “true” identification: A theory of enjoyable alterations pf players' self‐perception. Communication Theory, 19, 351–373. 10.1111/j.1468-2885.2009.01347.x

[icd2223-bib-0045] Krafft, K. C., & Berk, L. E. (1998). Private speech in two preschools: Significance of open‐ended activities and make‐believe play for verbal self‐regulation. Early Childhood Research Quarterly, 13(4), 637–658. 10.1016/S0885-2006(99)80065-9

[icd2223-bib-0046] Leach, J., Howe, N., & Dehart, G. (2015). ‘An earthquake shocked up the land!’ Children's communication during play with siblings and friends. Social Development, 24(1), 95–112. 10.1111/sode.12086

[icd2223-bib-0047] Leach, J., Howe, N., & DeHart, G. (2017). “I wish my people can be like the ducks”: Children's references to internal states with siblings and friends from early to middle childhood. Infant and Child Development, 26(5), e2015. 10.1002/icd.2015

[icd2223-bib-0048] Leach, J., Howe, N., & DeHart, G. (2019). A longitudinal investigation of siblings' and friends' features of connectedness and interaction quality during play. Early Education and Development, 30(6), 709–723. 10.1080/10409289.2019.1597589

[icd2223-bib-0049] Leekam, S. R., & Prior, M. (1994). Can autistic children distinguish lies from jokes? A second look at second‐order belief attribution. Journal of Child Psychology and Psychiatry, 35(5), 901–915. 10.1111/j.1469-7610.1994.tb02301.x 7962247

[icd2223-bib-0050] Leslie, A. M. (1987). Pretense and representation: The origins of “Theory of Mind”. Psychological Review, 94(4), 412–426. 10.1037/0033-295X.94.4.412

[icd2223-bib-0051] Liao, S., & Gendler, T. S. (2011). Pretense and imagination. Wiley Interdisciplinary Reviews: Cognitive Science, 2(1), 79–94. 26301915 10.1002/wcs.91

[icd2223-bib-0052] Lillard, A. S. (2013). Fictional worlds, the neuroscience of the imagination, and childhood education. In M. Taylor (Ed.), The Oxford handbook of the development of imagination. New York: Oxford University Press.

[icd2223-bib-0053] Lillard, A. S. (2014). The development of play. In R. M. Lerner Handbook of child psychology and developmental science. Hoboken, New Jersey: John Wiley & Sons, Inc. 10.1002/9781118963418.childpsy211

[icd2223-bib-0054] Longobardi, E., Spataro, P., Renna, M., & Rossi‐Arnaud, C. (2014). Comparing fictional, personal, and hypothetical narratives in primary school: Story gramma and mental state language. European Journal of Psychology of Education, 29(2), 257–275. 10.1007/s10212-013-0197-y

[icd2223-bib-0055] Mar, R. A. (2018). Stories and promotion of social cognition. Current Directions in Psychological Science, 27, 257–262. 10.1177/0963721417749654

[icd2223-bib-0056] Mar, R. A., & Oatley, K. (2008). The function of fiction is the abstraction and simulation of social experience. Perspectives on Psychological Science, 3(3), 173–192. 10.1111/j.1745-6924.2008.00073.x 26158934

[icd2223-bib-0057] Mar, R. A., Tackett, J. L., & Moore, C. (2010). Exposure to media and theory‐of‐mind development in pre‐schoolers. Cognitive Development, 25, 69–78. 10.1016/j.cogdev.2009.11.002

[icd2223-bib-0058] Meins, E., Fernyhough, C., Wainwright, R., Gupta, M. D., Fradley, E., & Tuckey, M. (2002). Maternal mind‐mindedness and attachment security as predictors of mind understanding. Child Development, 73(6), 1715–1726. 10.1111/1467-8624.00501 12487489

[icd2223-bib-0059] Ofcom . (2019). Children and Parents: Media Use and Attitudes.

[icd2223-bib-0004] Paine, A. L., Pearce, H., van Goozen, S. H. M., de Sonneville, L. M. J., & Hay, D. F . (2017). Late, but not early, arriving younger siblings foster firstborns’ understanding of second‐order false belief. Journal of Experimental Child Psychology, 166, 251–265. 10.1016/j.jecp.2017.08.007 PMC5714618 28946045

[icd2223-bib-0005] Paine, A. L., Hashmi, S., Roberts, S., Fyfield, R., & Hay, D. F . (2019a) Concurrent associations between mothers’ references to internal states and children's social understanding in middle childhood. Social Development, 28(3), 529–548. 10.1111/sode.12356 PMC6988506 32025110

[icd2223-bib-0006] Paine, A. L., Howe, N., Karajian, G., Hay, D. F., & DeHart, G . (2019b). H, I, J, K, L, M, N, O, PEE! Get it? Pee!': Siblings' shared humour in childhood. British Journal of Developmental Psychology, 1–18. 10.1111/bjdp.12277 30623983

[icd2223-bib-0060] Piaget, J. (1962). Play, dreams and imitation in childhood. London, England: Routledge & Kegan Paul.

[icd2223-bib-0061] Plowman, L., & Luckin, R. (2004). Interactivity, interfaces, and smart toys. Computer, 37(2), 98–100. 10.1109/MC.2004.1266302

[icd2223-bib-0062] Quiggle, N. L., Garber, J., Panak, W. F., & Dodge, K. A. (1992). Social information processing in aggressive and depressed children. Child Development, 63(6), 1305–1320. 10.1111/j.1467-8624.1992.tb01696.x 1446554

[icd2223-bib-0063] R Core Team . (2018). R: A language and environment for statistical computing. Vienna, Austria: R Foundation for Statistical Computing Retrieved from https://www.R-project.org/

[icd2223-bib-0002] Roberts, S., Fyfield, R., Baibazarova, E., van Goozen, S., Culling, J. F., & Hay, D. F. (2013) Parental speech at 6 months predicts joint attention at 12 months. Infancy, 18(S1), E1‐E15. 10.1111/infa.12018

[icd2223-bib-0064] Ruffman, T., Slade, L., & Crowe, E. (2002). The relation between children's and mothers' mental state language and theory‐of‐mind understanding. Child Development, 73, 734–751. 10.1111/1467-8624.00435 12038548

[icd2223-bib-0065] Scarlett, W. G., & Wolf, D. (1979). When it's only make‐believe: The construction of a boundary between fantasy and reality in storytelling. New Directions for Child Development, 6, 29–40. 10.1002/cd.23219790605

[icd2223-bib-0066] Singer, D. G., & Singer, J. L. (2005). Imagination and play in the electronic age. Cambridge, Massachusetts and London: Harvard University Press.

[icd2223-bib-0067] Singer, D. G., & Singer, J. L. (2013). Reflections on pretend play, imagination, and child development. American Journal of Play, 6(1), 1–14.

[icd2223-bib-0068] Slade, L., & Ruffman, T. (2005). How language does (and does not) relate to theory of mind: A longitudinal study of syntax, semantics, working memory and false belief. British Journal of Developmental Psychology, 23, 117–141. 10.1348/026151004X21332

[icd2223-bib-0069] Søndergaard, D. M. (2013). Virtual materiality, potentiality and subjectivity: How do we conceptualize real‐virtual interaction embodied and enacted in computer gaming imagination and night dreams? Subjectivity, 6(1), 55–78. 10.1057/sub.2012.23

[icd2223-bib-0070] Subrahmanyam, K., Kraut, R., Greenfield, P., & Gross, E. (2001). New forms of electronic media: The impact of interactive games and the internet on cognition, socialization, and behaviour. In D. G. Singer & J. L. Singer (Eds.), Handbook of children and the media (pp. 29–45). California, CA: Sage Publications, Inc..

[icd2223-bib-0071] Tompkins, V., Benigno, J. P., Kiger Lee, B., & Wright, B. M. (2018). The relation between parents' mental state talk and children's social understanding: A meta‐analysis. Social Development, 27(2), 223–246. 10.1111/sode.12280

[icd2223-bib-0072] Trawick‐Smith, J., Russell, H., & Swaminathan, S. (2010). Measuring the effects of toys on the problem‐solving, creative and social behaviours of preschool children. Early Child Development and Care, 181, 909–927. 10.1080/03004430.2010.503892

[icd2223-bib-0073] Turkle, S. (1997). Life on the screen: Identity in the age of the internet. New York, NY: Simon & Schuster.

[icd2223-bib-0074] Valkenburg, P. M. (2001). Television and the child's developing imagination. In D. G. Singer & J. L. Singer (Eds.), Handbook of children and the media (pp. 29–45). California, CA: Sage Publications, Inc..

[icd2223-bib-0075] Warton, D. I., & Hui, F. K. C. (2011). The arcsine is asinine: The analysis of proportions in ecology. Ecology, 92(1), 3–10. 10.1890/10-0340.1 21560670

[icd2223-bib-0076] Wolf, D., & Grollman, S. H. (1982). Ways of playing: Individual differences in imaginative style. In J. A. Damon (Ed.), The play of children: Current theory and research (pp. 46–63). New York, NY: Buffalo.

