# Supplementary material for: Seven‐year‐olds' references to internal states when playing with toy figures and a video game
Source: Infant Child Dev. 2021 Mar 9;30(3):e2223. doi: 10.1002/icd.2223 (PMC8404204; doi:10.1002/icd.2223)
Supplement: Supplementary file 1 — supplementary materials [file ICD-30-e2223-s001.pdf]

The Castell Arth Mawr Adventure Game

In this section, the narrative of the Castell Arth Mawr Game (CAMGame) is described, including a description of the possible choices, outcomes and speech from the characters (for a video demonstration of the CAMGame, visit https://youtu.be/SpixvsHypg8). The nature of the game allowed for children to explore or use their mallets in any way that they liked; however, for the purposes of this section, the description of the choices in relation to the use of the mallet are restricted to those recorded by the testers on the day as analysed in Hay and colleagues (2017).

When the game was loaded, experimenters demonstrated how to use the controller to move around, and how to look around. They then gave the children the following information: "*In this game, you can pretend that you are on a school trip to Castell Arth Mawr with your teacher and your friends. Some of the people might speak to you in the game, so it's important that you stop and listen carefully to everyone that talks to you, as they will tell you what to do and where to go in the game,*" (experimenters did vary in this script, but gave the same information).

The Beginning Level

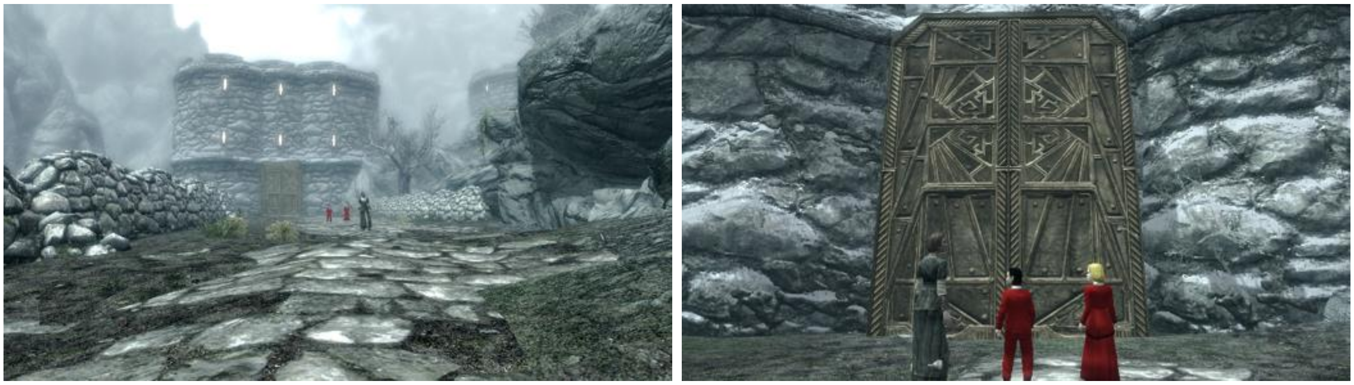

Figure S1. The beginning scene of the game. Figure S1a is on the left and S1b is on the right.

As the child moves the avatar forwards towards the teacher and the children wearing red sweatshirts (see Figure S1a), a voice is heard saying: *"Welcome to the Castell Arth Mawr adventure. To move forwards push the white button forwards. To move sideways, press the white button left or white. To look around, use the black button."* As the child approaches the teacher, she says: *"Hello there, I'm your teacher Mrs Williams, Castell Arth Mawr is just up ahead."* When the child continues forwards towards the children in the red sweatshirts from the red

school, the boy introduces them both: *"Hi! I'm Tom, this is Cerys. We're here to see Castell Arth Mawr."*

As the child walks forwards towards the golden door, the children wearing the red sweatshirts and the teacher can also be seen moving towards the door (see Figure S1b). When the child reaches the door, the teacher says: *"This is a door, to go through doors press the white button forwards."*

The Bottle Scene

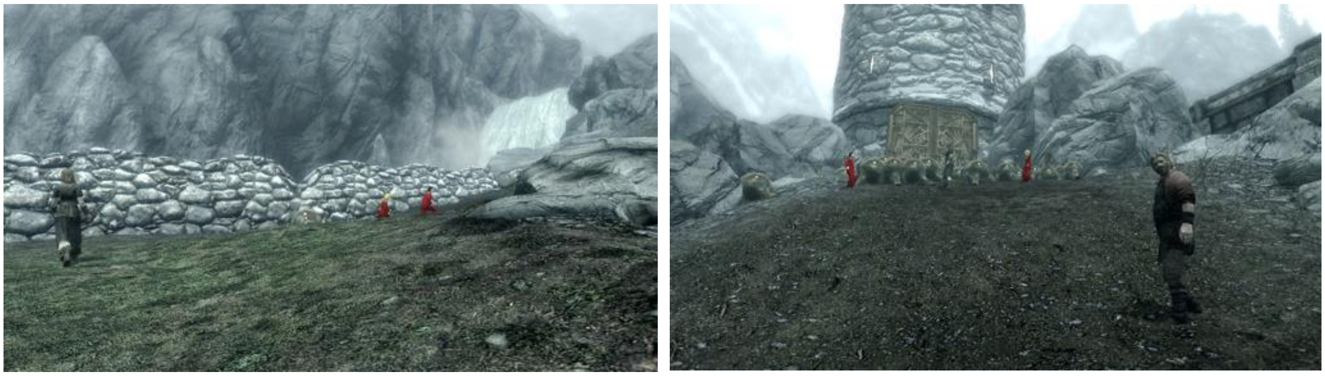

Figure S2. The second scene of the game.

After the child walks up to the door and goes through to the next level, the teacher and children from the red school walk towards a hill on the right, at the top of which the next gold door can be seen behind some bottles (see Figure S2). In front of the bottles, there is a man standing facing down the hill. As the child approaches the bottles, the man says: *"Look at these bottles, what a mess! You've been given a mallet you can use to help you get to the door. To get your mallet out, press the purple button. To swing the mallet, press the purple button again. To put the mallet away, press the yellow button."*

<u>CHOICE</u>: Use the mallet to hit the bottles and go to the door **<u>or</u>** walk through the bottles to the door.

The First Push Scene

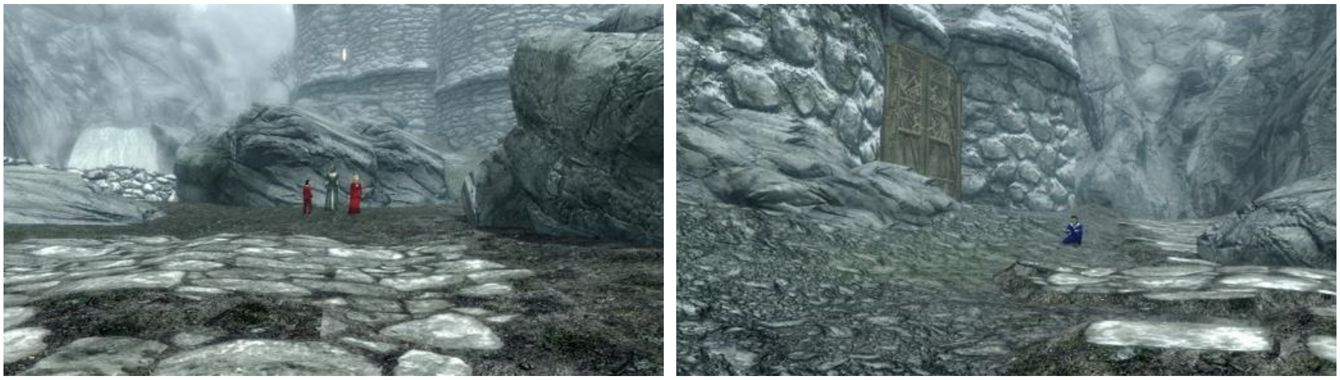

Figure S3. The third scene of the game.

After the child goes through the door and walks forward, the teacher says: *"If that mallet is in your hand, press the yellow button to put it away please."* In the distance, in front of the gold door leading to the next scene, there is a child from another school in a blue sweatshirt (see Figure S3).

As the child walks up the path, Tom says: *"Who's that,"* and Cerys says: *"He's from that blue school!"* As the child approaches the boy from the blue school and the door, the boy says: *"You red school loser,"* and the child is 'pushed' backwards.

<u>CHOICE</u>: Use the mallet to hit the boy from the blue school and go to the door **<u>or</u>** walk passed the boy from the blue school to the door.

The Castle Gates Scene

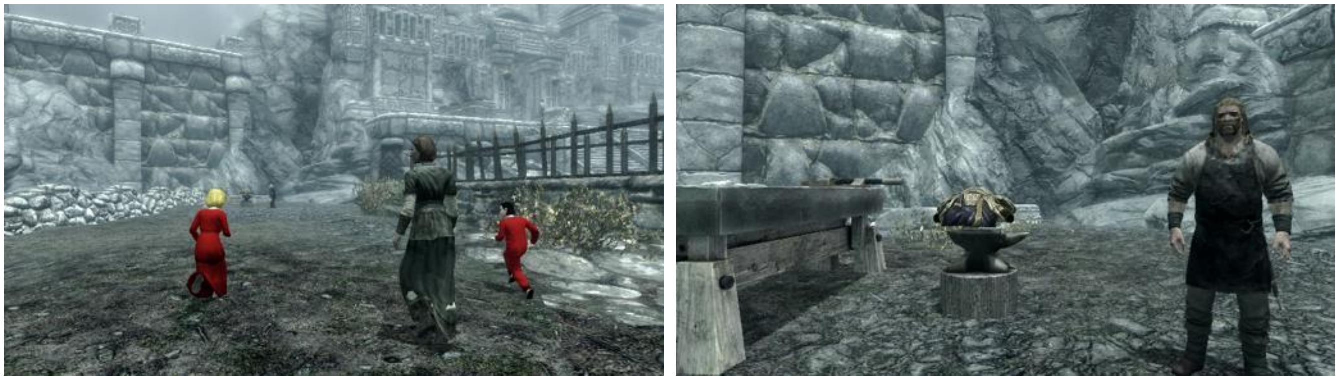

Figure S4. The opening of the Castle Gates Scene.

When the child goes through the door, there is a blacksmith standing next to an anvil and a workbench directly in front of the avatar (see Figure S4). When the child walks towards him,

he says: *"Hello there, I'm the castle blacksmith, could you help me? I see you have a mallet there, could you hit this piece of armour a few times? It's for a game later. To get your mallet out, press the purple button. To put it away press the yellow button."*

<u>CHOICE</u>: Use the mallet to hit the armour and continue passed the blacksmith **<u>or</u>** just continue passed the blacksmith.

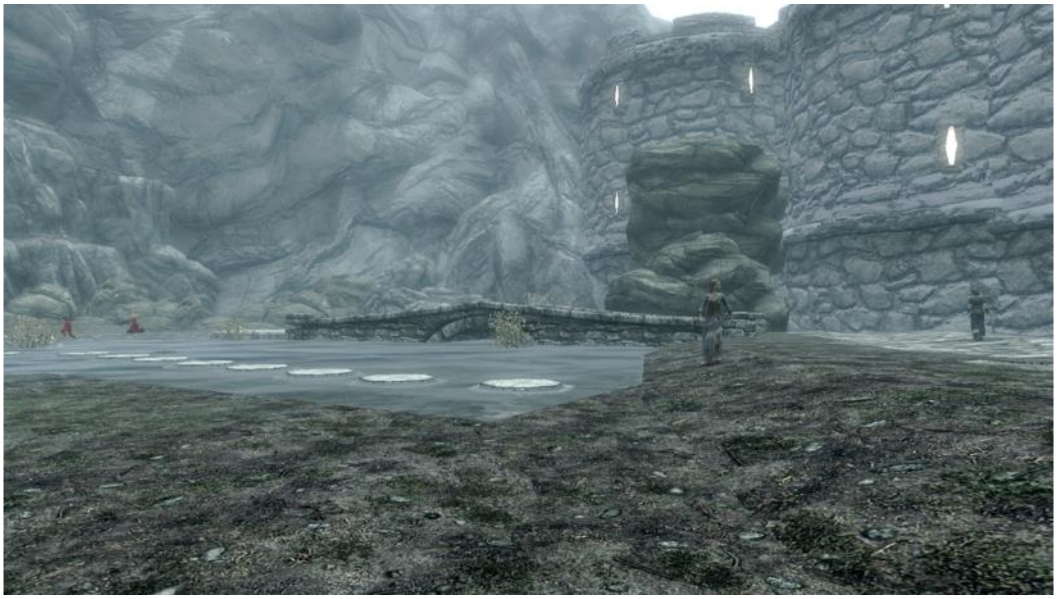

Figure S5. The stepping stones, bridge and river from the Castle Gates Scene.

Behind the blacksmith there is a lady standing next to some stepping stones leading across a river (see Figure S5). As the child walks towards her, she says: *"Hello there. Can you jump like me? Press the green button to jump. Try crossing these stepping stones. To move forward and jump, press the green button and move forwards."* The child can either cross the river by jumping across the stepping stones, or walk across the bridge that is behind the lady.

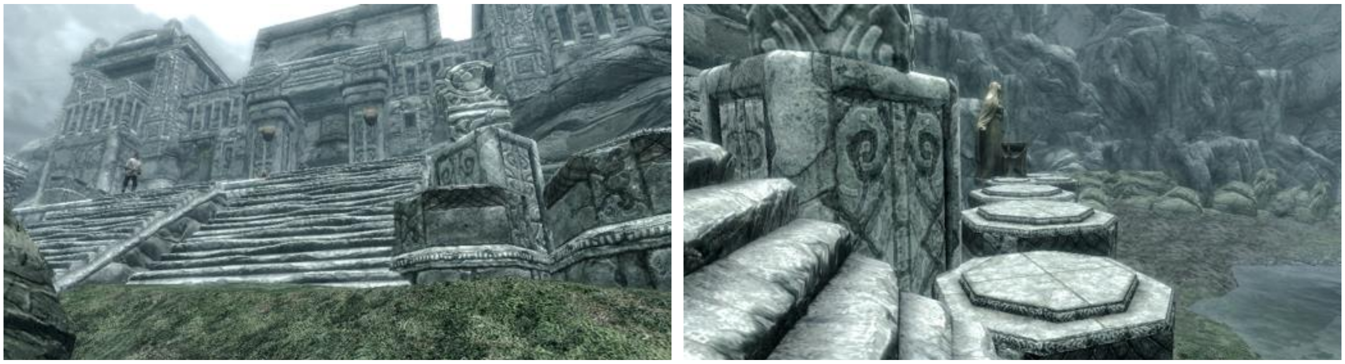

Figure S6. The stairs to the castle gates and location of the magic statue in the Castle Gates Scene. Figure S6a is on the left and S6b is on the right.

On the other side of the river there are some stairs leading up to the castle gates, and when the child reaches the stairs, the teacher says: *"There it is, Castell Arth Mawr!"* (see Figure S6a). When the child goes up the stairs and to the castle gates, a voice can be heard that says: *"These are magic gates, they only open when you talk to the magic statue. You'll have to go looking for it."* The magic statue can be found by the child across from the stairs (see Figure S6b). When the child approaches the magic statue, a voice can be heard saying: *"Well done, you have found the magic statue. The magic gates are now open."* The child can then walk back to the gates and through the door into the castle.

The Castle Main Hall Scene

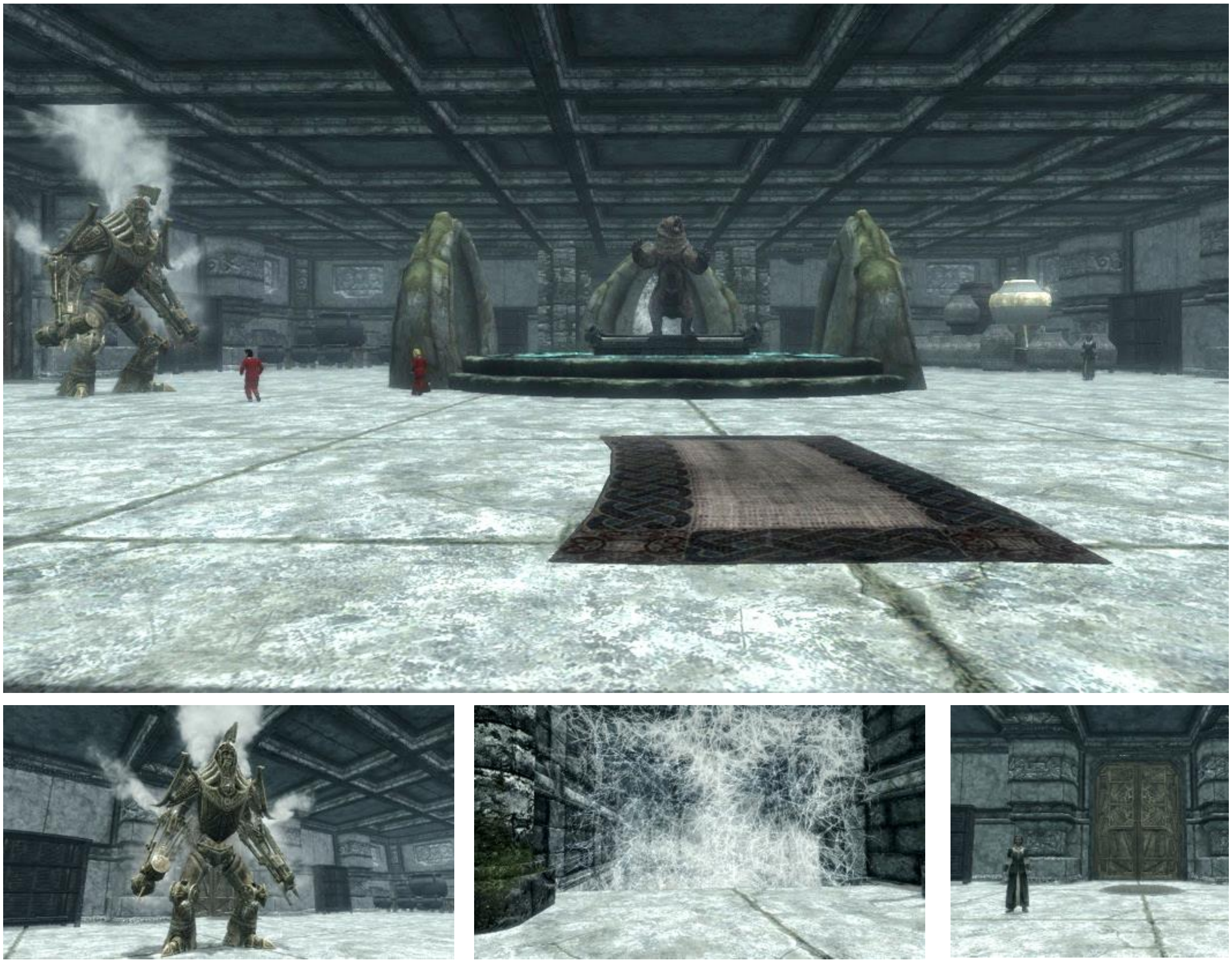

Figure S7. The Castle Main Hall Scene

When the child enters the main hall of the castle, there is a bear on a statue directly in front of them (see Figure S7). To the left of the bear statue, there is a golden robot standing in front of a door emitting steam; behind the bear statue there are some cobwebs leading down some stairs to a barely visible door; and to the right of the bear statue the teacher is standing in front of another door (see Figure S7).

If the child approaches the golden robot, he says: *"Hello there! I am the Castle Guardian! Go through this door and I shall guide you!"*

If the child approaches the teacher, she says: *"Are you ready? Okay let's go this way."*

If the child uses the mallet to hit the bottles or furniture in this area, the teacher says: *"Don't do that please!"* which prompts a choice.

<u>CHOICE</u>: Use the mallet to hit the bottles or furniture and stops when asked **<u>or</u>** uses the mallet to hit the bottles or furniture and does not stop after being asked to.

<u>CHOICE</u>: Goes through the door by the Castle Guardian **<u>or</u>** goes through the door behind the cobwebs **<u>or</u>** goes through the door by the teacher.

The Broken Bridge Scene

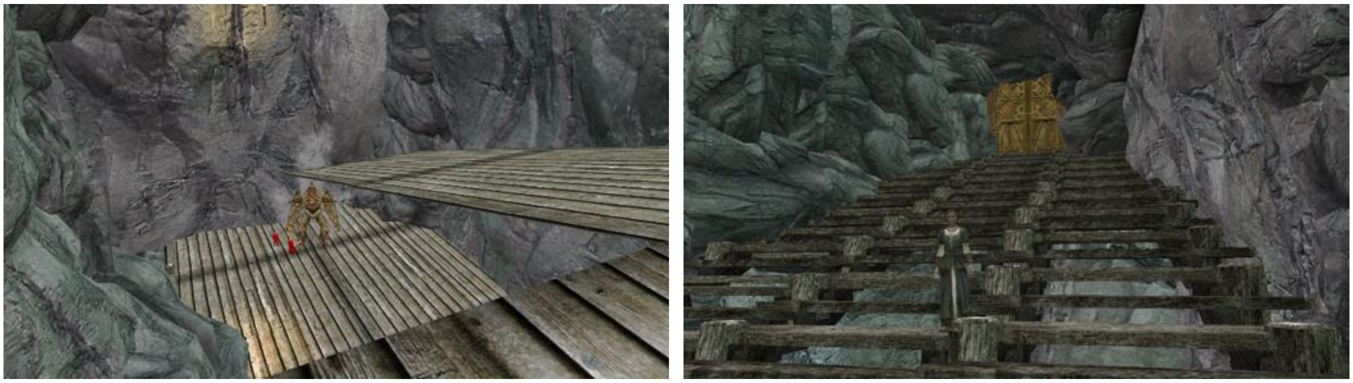

Figure S8. The Broken Bridge Scene. Figure S8a is on the left and S8b is on the right.

As soon as the child enters the next scene, a ledge can be seen in front of them and Cerys can be heard saying: *"I'm here with Tom, we both fell down here!"* (See Figure S8a). Behind the child, the teacher can be seen standing on the stairs in front of a golden door (see Figure S8b).

If the child approaches the teacher, she says: *"Okay, you two go on ahead. We'll find another way round."*

If the avatar walks forwards off of the ledge, it will land on the platform with the other children from the red school (and the Castle Guardian if they went through his door in the previous scene) and Cerys will say: *"Phew you made it! Let's go this way."* A golden door is visible down some stairs from this platform.

<u>CHOICE</u>: Go through the door up the stairs behind the teacher **<u>or</u>** fall down off of the ledge and go through the door that is accessible from the platform.

The Storyteller Scene

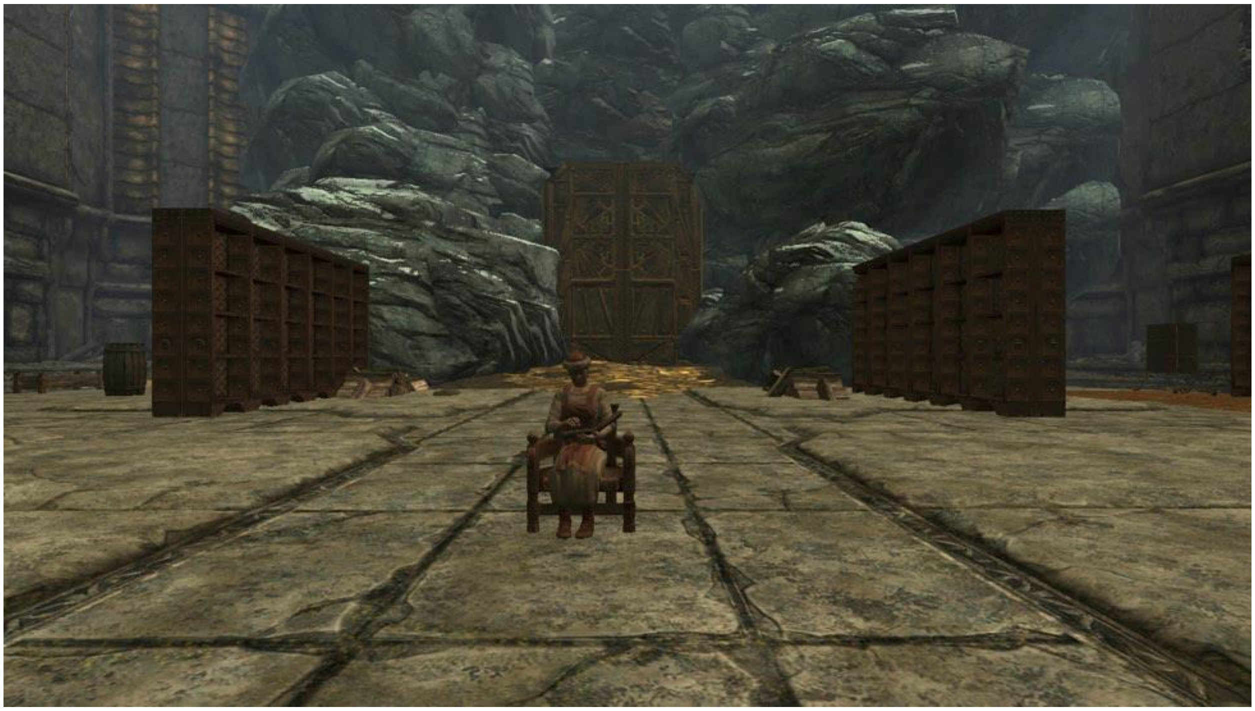

Figure S9. The Storyteller Scene

When the child goes through either door and enters into this scene, an elderly character, the Storyteller, can be seen sitting in a chair reading a book in front of the golden door (see Figure 9). As the child walks towards the storyteller, she says: *"Hello children I am the Storyteller! This was the castle of the Bear King, before he left he buried treasure in the caves! If you're quick you might find it, before those blue school kids do."*

<u>CHOICE</u>: Use the mallet to hit the storyteller **<u>or</u>** walk past the storyteller towards the door.

As the child approaches the door to the next scene, the Storyteller says: *"Before you go, could you help me? Could you hit that woodpile with your mallet? I need it for later to warm up."*

CHOICE: Use the mallet to hit the woodpile and go through the door **or** just go through the door.

The Cave Scene

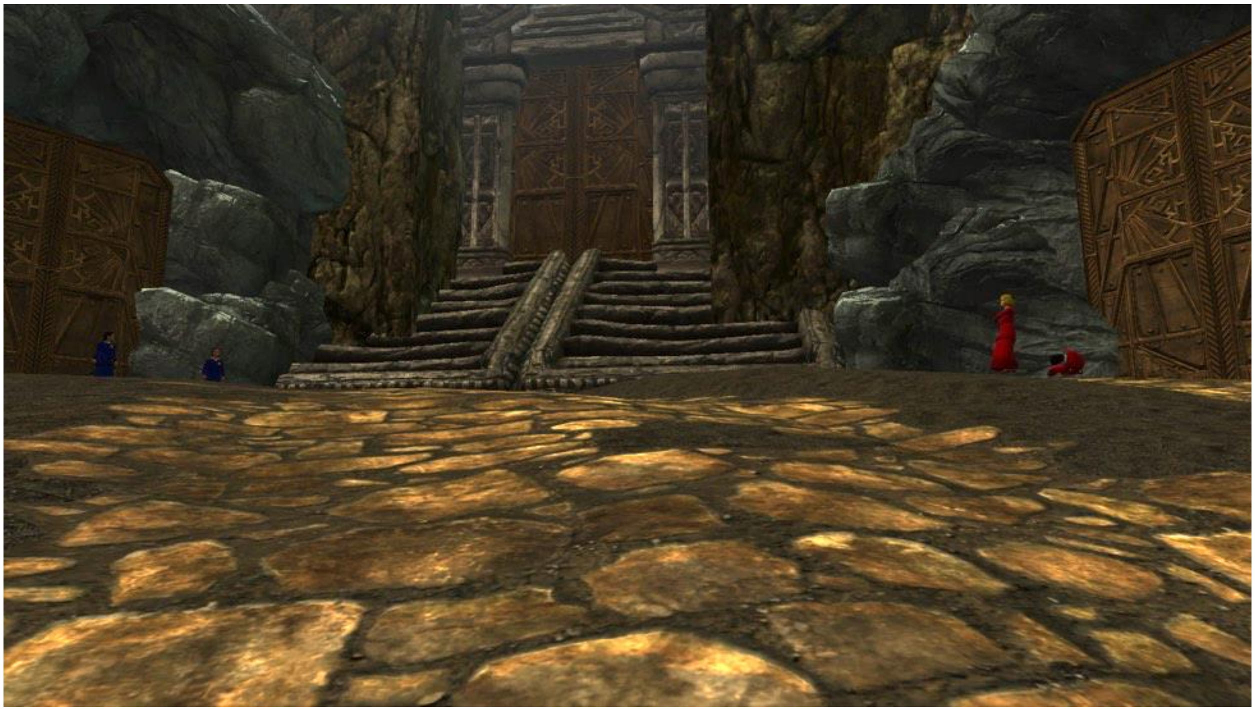

Figure S10. The Cave Scene.

As soon as the avatar enters into this scene, it is pushed backwards and the girl from the blue school can be heard saying: *"You red school loser!"* followed by the boy from the blue school saying: *"I pushed you and that means go away."* In front of the child, the blue school children can be seen standing in front of a door on the left; there is a door up some stairs straight ahead; and the red school children can be seen in front of a door on the right (see Figure S10). Only the door that at the top of the stairs will take the child to the next scene. As the child walks forwards, Tom can be heard saying: *"Oww! They pushed me!"*

If the child approaches the red school children, Tom says: *"Oh thank you, I'll be okay,"* and Cerys says: *"I'll stay with Tom. We're going to try this door, you try the door up the stairs."*

If the child approaches the blue school children, the boy from the blue school says: *"Yeah we pushed you and your friends, so what? We're gonna get the treasure before you."*

CHOICE: The child approaches the red school children and stops **or** does not.

CHOICE: The child uses the mallet to hit the children from the blue school **or** does not.

Alternatively, the child can do neither of these actions and proceed to the next scene by going through the golden door at the top of the stairs.

The Ditch Scene

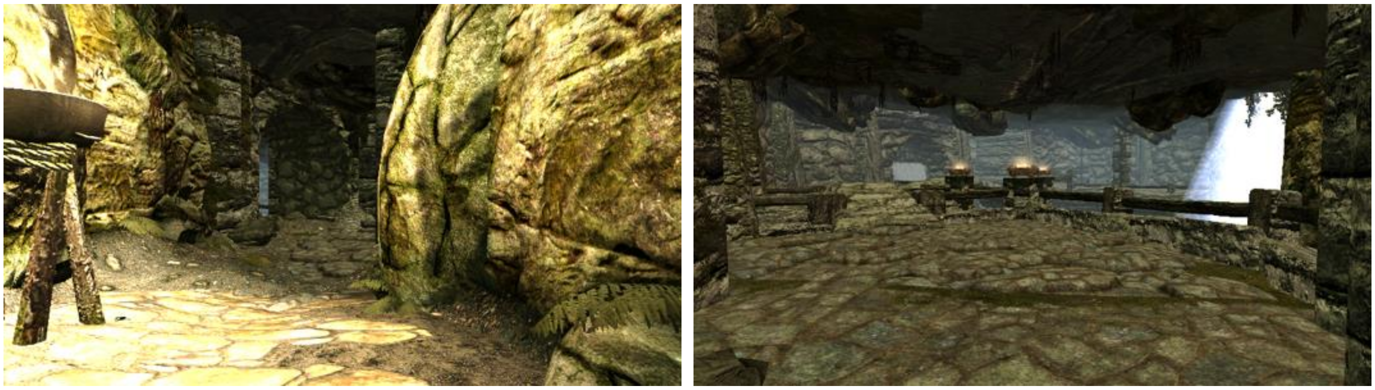

Figure S11. The beginning of the Ditch Scene.

When the avatar goes through the door, it is presented with a dark corridor to walk forwards through (see Figure S11). As they walk forwards, Cerys can be heard saying: *"Hey! What are you doing you meanies?"* The boy from the blue school can then be heard saying *"Haha, you won't find the treasure now losers."* When the child reaches the end of this corridor, two bridges can be seen to the left where they need to cross (see Figure S11).

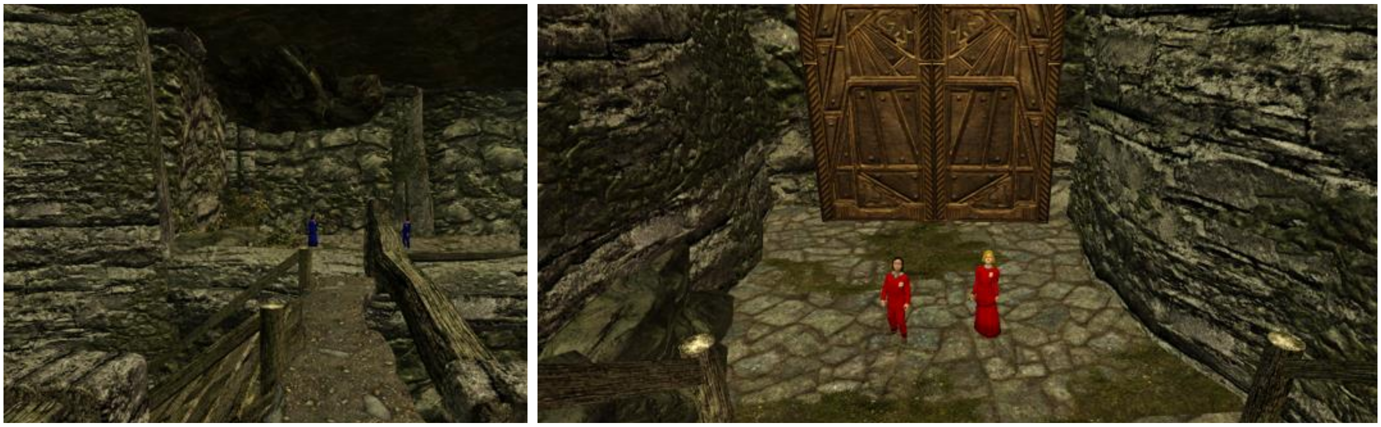

Figure S12. The end of the Ditch Scene.

When the child reaches the middle of the second bridge, the blue school children can be seen directly ahead (see Figure S12) and Tom can be heard saying *"Those mean bullies pushed us down here!"* As the child continues moving forwards, the girl from the blue school says: *"Oh look, it's stupid again."* At the end of the bridge there are some stairs leading down to where the red children are who are standing in front of the door to the next scene (see Figure S12).

CHOICE: The child uses the mallet to hit the children from the blue school **or** does not.

CHOICE: The child approaches the red school children and stops **or** does not.

Alternatively, the child can do neither of these actions and proceed to the next scene by going through the golden door.

The Racing to the Treasure Scene

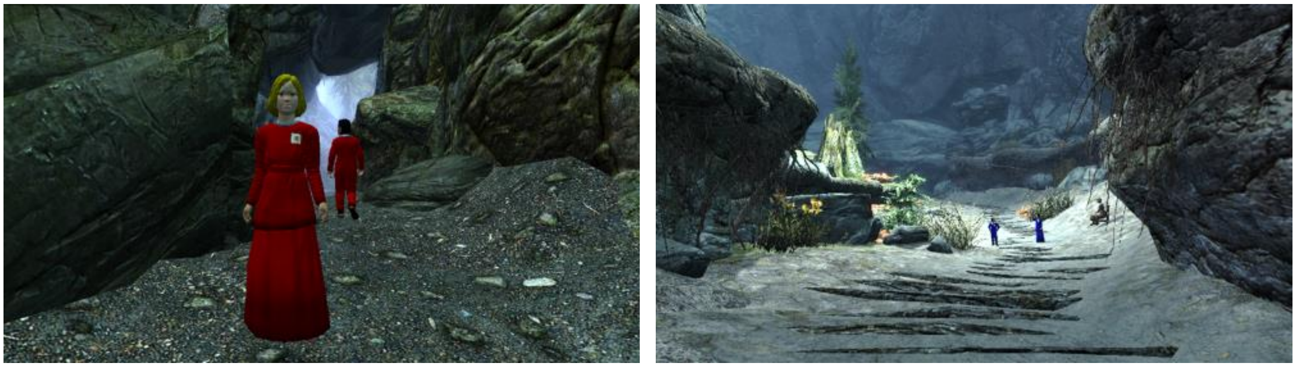

Figure S13. The beginning of the Racing to the Treasure Scene.

As the avatar enters this scene, it is presented with a dark corridor with the children from the red school (see Figure S13). When it reaches the end of the corridor, it is presented with a large cavern with a winding path surrounded by foliage and waterfalls (see Figures S13 & S14). Ahead along the path, the Storyteller is sat on a chair next to the children from the blue school and when she is approached she says: *"Oh hello again children, I hope you've all been getting along. The treasure's not far from here, you'll have to go looking for the magic statue. It's somewhere around here."*

CHOICE: The child uses the mallet to hit the children from the blue school **or** does not.

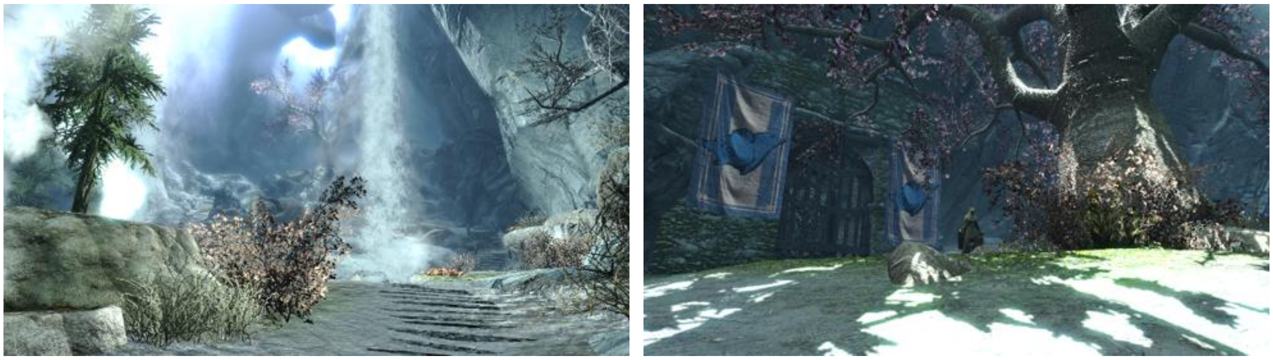

Figure S14. The Racing to the Treasure Scene

Along the path to the door there is a golden door which the child cannot get to and a picturesque surrounding to explore. When the child reaches the end of the path, there is a large tree in front of the gates to the castle with the magic statue next to the gates (see Figure S14). As the child approaches the gates, the boy from the blue school says: *"This stupid door won't open,"* and the girl from the blue school says: *"This statue won't do anything."* When the child approaches the statue, the gates can be heard to open and the child can proceed through the door.

The Bear King Scene

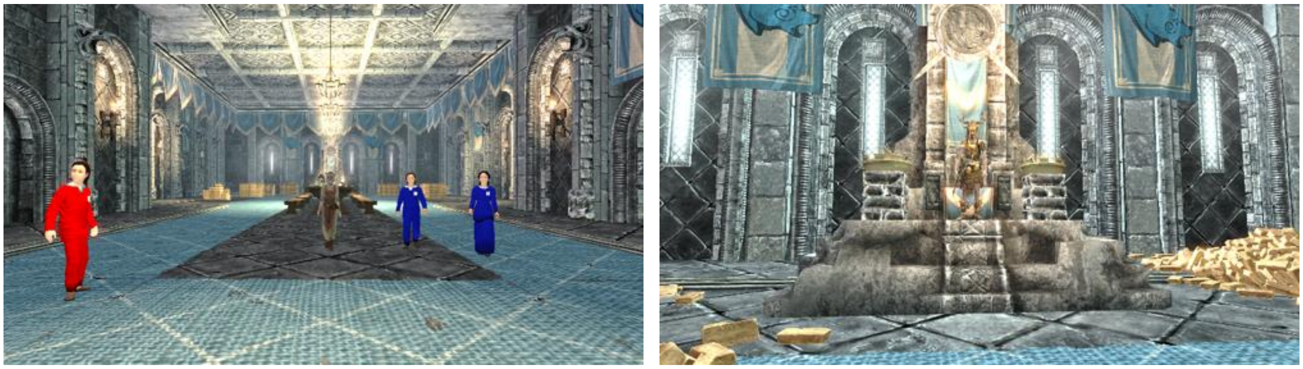

Figure S15. The Bear King Scene.

As soon as the child proceeds through the door, which can take a while to load, the Storyteller and children from both schools are directly in front (see Figure S15). The Storyteller immediately says: *"Yes, well done, you have found the treasure! And this is the Bear King!"* On the other end of the room, the Bear King is standing by a throne (see Figure S15) and when approached says: *"Hello children! I am the Bear King! Well done, you have found my treasure. The game is over."*
